# The Sugar Transporter family in wheat (*Triticum aestivum*. L): genome-wide identification, classification, and expression profiling during stress in seedlings

**DOI:** 10.7717/peerj.11371

**Published:** 2021-05-04

**Authors:** Hongzhan Liu, Chaoqiong Li, Lin Qiao, Lizong Hu, Xueqin Wang, Junsheng Wang, Xianle Ruan, Guangyu Yang, Guihong Yin, Chunping Wang, Zhongke Sun, Keshi Ma, Lili Li

**Affiliations:** 1 Zhoukou Academy of Agricultural Sciences, Zhoukou, Henan, China; 2 College of Life Science and Agronomy, Zhoukou Normal University, Zhoukou, Henan, China; 3 College of Agronomy, Henan Agricultural University, Zhengzhou, Henan, China; 4 Key Laboratory of Plant Genetics and Molecular Breeding, Zhoukou Normal University, Zhoukou, Henan, China; 5 College of Agronomy, Henan University of Science and Technology, Luoyang, Henan, China

**Keywords:** Wheat, Gene family, Sugar transporter, Syntenic maps, qRT-PCR

## Abstract

The sugar transporter protein (STP) plays a crucial role in regulating plant growth and stress tolerance. We performed genome-wide identification and expression analysis of the STP gene family to investigate the STPSs’ potential roles in the growth of wheat seedlings under stress. Here, a total of 81 TaSTP genes containing the Sugar_tr conserved motif were identified within the wheat genome. Bioinformatic studies including phylogenetic tree, chromosome position, and tandem repeat were performed to analyze the identified genes. The 81 TaSTP genes can be classified into five main groups according to their structural and phylogenetic features, with several subgroups, which were located separately on chromosomes A, B, and D. Moreover, six gene clusters were formed with more than three genes each. The results of three comparative syntenic maps of wheat associated with three representative species suggested that STP genes have strong relationships in monocots. qRT-PCR analysis confirmed that most TaSTP genes displayed different expression profiles after seedlings were subjected to six days of different stress (10% PEG6000, 150 mM NaCl, and their combination, respectively), suggesting that these genes may be involved in regulating plant growth and stress tolerance. In conclusion, 81 TaSTP genes were identified and their expressions changed under stress, indicating TaSTP’s potential roles in wheat growth monosaccharide distribution is regulated.

## Introduction

Sugar is used as a universal energy source in higher land plants, playing important roles in cell development, signal transmission, and osmotic homeostasis under certain abiotic stress conditions (Lastdrager, Hanson & Smeekens, 2014; Rolland, Baena-Gonzalez & Sheen, 2006). To date, it has been established that sugars produced by photosynthesis are distributed mainly as sucrose through the phloem to other parts of the plant. Sucrose is transported over long distances to the sink organs, some of which is unloaded directly to the sink organs via the symplast. Apoplastic sugar is the breakdown product of sucrose into glucose and fructose by sucrose invertase, and is taken in by transmembrane absorption mediated by sugar transporters (STP) before entering the sink cells (Bush, 1999; Ludewig & Flügge, 2013; Paulsen, Custódio & Pedersen, 2019). Sugar is typically transported across the plasma membrane or the vacuolar membrane by sugar transporters through active transport mechanisms or accelerated passive transport. In apoplastic loading, transmembrane transport is carried out by the sucrose transporter (SUT) or sugar carrier (SUC) in the plasma membrane (Braun & Slewinski, 2009). In the process of apoplastic unloading, invertase catalyzes the irreversible hydrolysis of sucrose into glucose and fructose, which is then unloaded by monosaccharide transporters (MST). Invertase and MST play a key role in this mode of transport. In sugarcane, eighty percent of the carbon fixed in leaves at midday is exported immediately (Du et al., 2000). Sucrose is the main form of long-distance carbohydrate transport in higher plants, while SUT, invertase, and MST are important in the loading and unloading of sugar molecules (Slewinski, Meeley & Braun, 2009; Zhang, Zhang & Liu, 2015; Doidy, Vidal & Lemoine, 2019). Sugar transport in plants is an important research topic and has economic implications (Hedrich, Sauer & Neuhaus, 2015). A key element for plant growth and development is the distribution of sugars between assimilate-exporting source tissues and sugar-consuming sink tissues; MST from the sugar transporter family contribute to the uptake of sugars into sink cells (Rottmann et al., 2018). More than 50 MSTs were identified in *Arabidopsis*. As a part of MST gene family, the STP subfamily is comprised of 14 monosaccharide/H+ symporters (Schofield et al., 2009). The STP subfamily encodes H^+^-symporting monosaccharide transporters, which are able to transfer diverse hexoses (e.g., glucose, mannose) and/or pentoses (e.g., xylose) but not sucrose (Rottmann et al., 2018). Almost all STPs are a high affinity hexose transporter with specific expression in tissues, indicating that one of its main functions is to build up the sink required for photosynthate redistribution (Büttner, 2007; Schofield et al., 2009). This process plays a key role in maintaining source/sink characteristics and hormonal signals, as highlighted in the case of abiotic or biotic stresses (Balibrea Lara et al., 2004; Roitsch & González, 2004).

The plant STP protein commonly contains 12 structurally-conserved transmembrane domains (a large loop located in the cytoplasm in the middle of the sequence divides the whole protein into two parts; each contain six transmembrane domains) (Bush, 1993; Yan, 2013). Since the first STP gene was cloned from Chlorella (Sauer & Tanner, 1989), multiple STP genes have been identified by genome-wide identification with the rapid development of whole-genome sequences in various plants, such as *Arabidopsis* (Büttner, 2010), *Vitis vinifera* (Afoufa-Bastien et al., 2010), tomato (*Solanum lycopersicum*) (Reuscher et al., 2014), rice (*Oryza sativa*) (Johnson & Thomas, 2007), cassava (*Manihot esculenta*) (Liu et al., 2018b), *Zea mays* (Kong et al., 2019), pear (*Pyrus bretschneideri Rehd*) (Li et al., 2015), sweet oranges (*Citrus sinensis*) (Zheng et al., 2014), and rapeseed (*Brassica napus* L.) (Zhang et al., 2020). In *Arabidopsis*, a total of 14 STP genes have been discovered to date, using a database search. These STP family genes exhibit different expression patterns and transport functions during plant growth and development and respond to stress processes (Büttner, 2010). For example, *AtSTP1* is reported to be abundant in seeds during imbibition and in seedling roots; moreover, transport by *AtSTP1* plays an important role in very high concentrations of exogenous sugar (Sherson et al., 2010). *AtSTP2* expression has been detected at the early stages of gametophyte development, specifically beginning at callose degradation and microspore release. Pollen-specific expression of *AtSTP6*, *AtSTP9* and *AtSTP11* has been shown during pollen germination and pollen tube growth (Schneidereit, Scholz-Starke & Büttner, 2003; Scholz-Starke, Büttner & Sauer, 2003; Schneidereit et al., 2005). Some reports have stated that AtSTP4 is mainly expressed in sink tissues such as roots and in mature leaves infected by fungi (Endler et al., 2006; Fotopoulos et al., 2003). Recent studies have shown that STP7 is highly expressed in tissues with cell wall transition, suggesting that STP7 may contribute to sugar uptake and is then recycling in the cell wall. STP8 and STP12 are highly expressed in the reproductive organs, and their protein products may contribute to the intake of sugar in the pollen tube and embryo (Rottmann et al., 2018).

In rice, the OsMST3 mRNA is detectable in leaf and roots (especially the sclerenchyma cells) as well as in the xylem in the root, which indicates that OsMST3 is involved in the accumulation of monosaccharides required for cell wall synthesis. Furthermore, STP genes also respond to stress from the environment, such as wounding or pathogen attack (Liu et al., 2018b). Moreover, drought stress has been reported to increase root length in the genotypes Gallagher, TAM111, and Yumar in wheat (Djanaguiraman et al., 2019).

Wheat is a globally important crop that supplies protein, vitamins, and minerals to humans. Wheat has a hexaploid A, B, and D genome (Brenchley et al., 2012). This species has a large 17 Gb genome, yet many STP genes remain undiscovered. STP genes play an important role in plant growth and development (Mathieu et al., 2018), so the identification of these gene families through genomic databases is important. We conducted an in-depth genome-wide analysis of the *TaSTP* gene family from the wheat genome (*International Wheat Genome Sequencing Consortium: IWGSC*) and included *TaSTP* gene models, genomic structures, phylogenetic relationships, chromosome locations, Ka/Ks ratios, and other bioinformatic analyses. Using qRT-PCR, we also analyzed the expression patterns of TaSTP genes in seedlings after 6 days of stress in NaCl, PEG6000, and NaCl+PEG6000. Our results will establish a solid foundation for further research on the functional roles of STP genes in wheat.

## Materials and Methods

### Plant materials

The wheat cultivar Zhoumai 36 was germinated and grown in a tissue culture room at Zhoukou Normal University. The light conditions were set to dark for 12 h and light for 12 hours at 25–30 °C. Wheat seedlings were watered once every three days with Hoagland’s nutrient solution. The stress treatment was performed when seedlings grew to the second-true leaf stage. Wheat seedlings were divided into four groups with 100 seedlings per group. Seedlings from the first group to the third group were treated with 10% PEG6000 solution, 150 mmol L^−1^NaCl, and 10% PEG6000 solution+150 mmol L^−1^NaCl, respectively. The other group was left untreated as a control check (CK). Furthermore, different seedling tissues, such as leaves, stems, and roots, were collected to prepare the mixed cDNA template, which was used for *TaSTP* gene cloning. All fresh materials were quickly frozen in liquid nitrogen and stored in a −80 °C refrigerator for total RNA isolation.

### Identification and characteristics of the TaSTP genes in wheat

The consensus protein sequences (PF00083) from the Sugar_tr hidden Markov model (HMM) were downloaded from Pfam 31.0 to identify the STP genes in wheat (https://pfam.xfam.org/family/PF00083). Then, the whole-genome data of wheat (*IWGSC*) and *Brachypodium distachyon* (BD) were obtained from the International Wheat Genome Sequencing Consortium and Phytozome v12.1 website. The HMM profile was used as a query to identify all STP-containing sequences in wheat and BD by searching against the downloaded genome with an *E*-value of <1e^−5^. Furthermore, all candidate TaSTPs were verified using Pfam and SMART (http://smart.embl-heidelberg.de/) to confirm that they contained the core domains. All potentially redundant TaSTP sequences and sequences lacking a part of STP domain or complete STP domain were discarded based on the sequence alignments generated by ClustalX software. Finally, a total of 81 STP protein sequences, which were encoded by 81 genes in the same subgroup as that in *Arabidopsis thaliana,* were identified in the wheat genome.

### Phylogenetic analysis

For the phylogenetic tree of *TaSTP*, 14 *Arabidopsis* AtSTPs, eight rice STP protein sequences, six brachypodium distachyon STP protein sequences, eight foxtail millet STP protein sequences, 20 cassava STP protein sequences, 20 *Ricinus communis* STP protein sequences, and 81 full-length TaSTP protein sequences were aligned using ClustalX 2.1 (http://clustalx.software.informer.com/2.1/). A neighbour-joining (N-J) tree was constructed with the alignments using MEGA 7.0 software (Kumar, Stecher & Tamura, 2016) with bootstrap analysis from 1,000 replicates. The STP genes were named *TaSTP1* to *TaSTP81* according to their positions on the chromosomes.

### Gene structure analysis and motif detection

The coding sequences (CDS) and genomic sequences of the STP genes were obtained from the International Wheat Genome Sequencing Consortium (http://plants.ensembl.org/Triticum_aestivum/Info/Index). By comparing the coding sequences and their chromosomal genomes, the exon-intron structures of the TaSTP family were generated using the Gene Structure Display Server website (http://gsds.gao-lab.org/). The MEME motifs of the predicted TaSTP proteins were identified using MEME suite 5.0.1 (http://meme-suite.org/tools/meme). Subsequently, these files were optimized using TBtools (Chen et al., 2018).

### Homologous gene pairs and synteny analysis

The Ka/Ks ratios of gene pairs in duplication blocks were calculated using DnasP v6.0 and performed as described by Cao et al. (2016). The parameters of the sliding window analysis of the Ka/Ks ratios were set as follows: window size, 200 bp; step size, 25 bp. Moreover, TaSTP duplications were performed using the MCScanX program in the Bio-Linux system. In brief, with the BLAST outputs, the file of the gene positions, and the use of the MCScanX program, the TaSTP genes were classified into different types of duplications. We used Circos software to construct a schematic diagram (Krzywinski et al., 2009; Liu et al., 2018b) to exhibit the synteny relationship of the orthologous TaSTP genes obtained from wheat and other selected species. The syntenic analysis maps were constructed using the Dual Systeny Plotter software (https://github.com/CJ-Chen/TBtools).

### Expression profiles of the TaSTP genes in the RNA-Seq data

Public transcriptome data were downloaded from the website http://www.wheat-expression.com/. Subsequently, all TPM data were log10 transformed, which was detailed in a previous study (Yue et al., 2018), and a heat map was created using TBtools.

### Network interaction and GO analysis

The protein interaction network analysis of the TaSTPs was conducted using the STRING v10.5 database (http://www.string-db.org) based on the orthologous genes between *Arabidopsis* and wheat. GO analysis was conducted by GOToolBox (http://genome.crg.es/GOToolBox/) and visualized with Tbtools.

### RNA isolation and validation analysis

Total RNA was extracted using the Trizol method (TriQuick Reagent, Solarbio, China), denatured in agarose gel (1%), and stained with SolarGelRed (Solarbio, China) to check the quality of the mixed RNA. The first-strand cDNAs were synthesized using a PrimeScript RT reagent kit (Takara Bio, Tokyo) according to the manufacturer’s instructions and stored at −20 °C. Specific primers were designed using Beacon Designer software version 8.13 (Premier Biosoft International, Palo Alto, CA, USA) for the quantitative real-time polymerase chain reaction (qRT-PCR); the primer sequence details are provided in Table S1. Detailed information on the qRT-PCR protocol was described in a previous study (Liu et al., 2018a).

**Figure 1:**
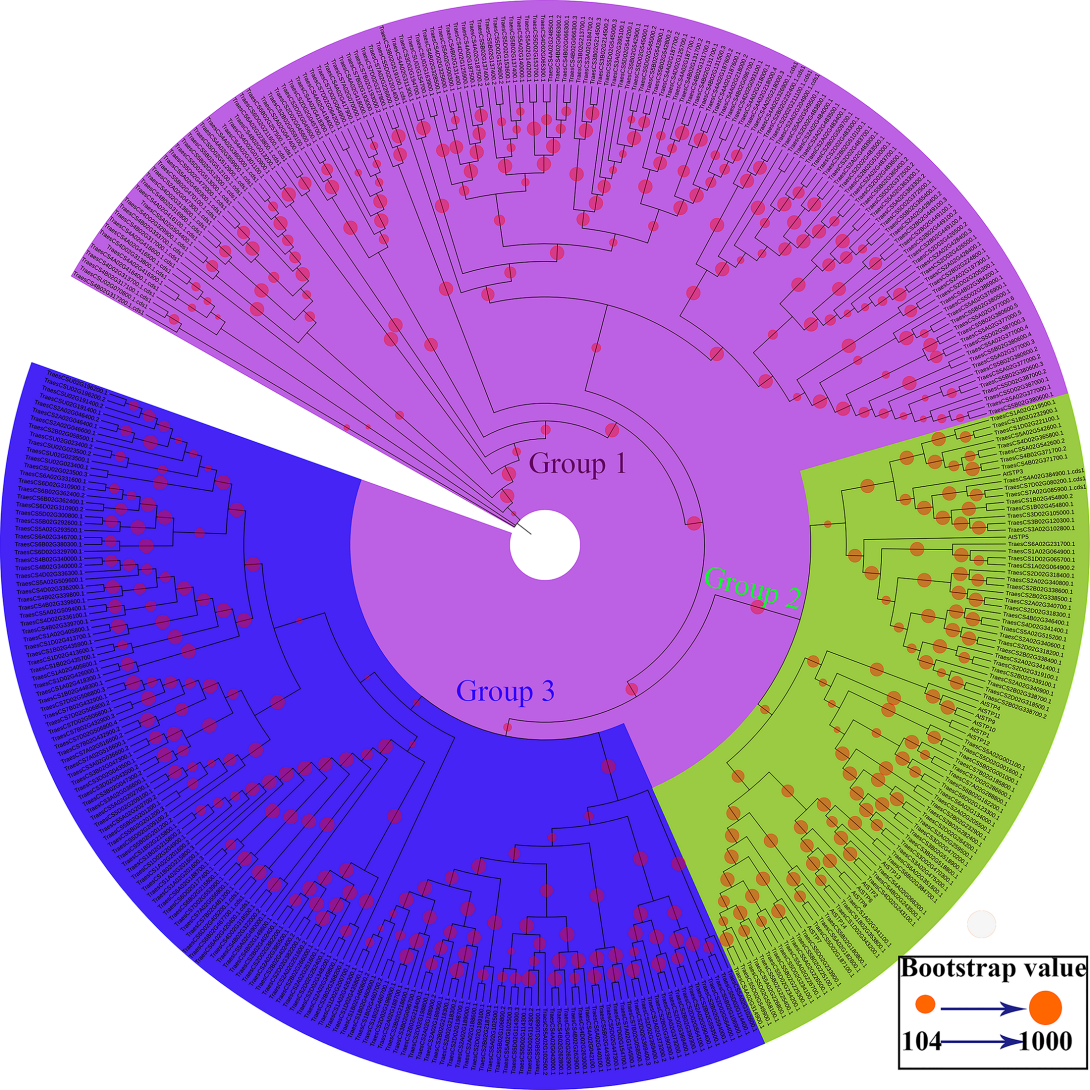
Phylogenetic relationships and subfamily designations of the STP proteins from wheat and *Arabidopsis thaliana*. An evolutionary tree was formed by the phylogenetic relationships of 390 predicted TaSTP proteins and *Arabidopsis thaliana* (*AtSTP1*-*AtSTP14*) proteins with 1,000 bootstrap replicates by MEGA v7.0. The subgroups containing STP proteins (*AtSTP1*-*AtSTP14*) from *Arabidopsis thaliana* are marked with green circle and black lines. There are 81 genes in wheat in the same group as that of *Arabidopsis thaliana*. The new names and accession numbers are shown in Table 1.

**Table 1:** Information about the TaSTP genes in wheat.

Gene Name	Gene Locus	CDS Length (bp)	AA^a^	MW^b^ (kDa)	pI^c^	TMD^d^
*TaSTP1*	TraesCS1A02G064900.1	1578	525	56.57	9.36	12
*TaSTP2*	TraesCS1A02G064900.2	1428	475	51.95	9.60	10
*TaSTP3*	TraesCS1A02G219500.1	1533	510	55.98	9.09	12
*TaSTP4*	TraesCS1A02G341100.1	1515	504	56.26	9.18	10
*TaSTP5*	TraesCS1B02G232900.1	1566	521	57.41	9.11	11
*TaSTP6*	TraesCS1B02G353800.1	1536	511	56.25	9.17	9
*TaSTP7*	TraesCS1B02G454800.1	1515	504	54.49	9.16	10
*TaSTP8*	TraesCS1B02G454800.2	1515	504	54.59	9.16	10
*TaSTP9*	TraesCS1D02G065700.1	1575	524	56.64	9.01	10
*TaSTP10*	TraesCS1D02G221100.1	1566	521	57.26	9.11	12
*TaSTP11*	TraesCS1D02G343200.1	1536	511	56.24	9.17	10
*TaSTP12*	TraesCS2A02G205500.1	1581	526	57.45	9.20	11
*TaSTP13*	TraesCS2A02G259500.1	1554	517	56.29	9.57	11
*TaSTP14*	TraesCS2A02G340600.1	1533	510	54.60	9.53	11
*TaSTP15*	TraesCS2A02G340700.1	1545	514	55.48	9.61	11
*TaSTP16*	TraesCS2A02G340800.1	1548	515	56.15	9.74	12
*TaSTP17*	TraesCS2A02G340900.1	1524	507	54.26	9.51	11
*TaSTP18*	TraesCS2A02G341400.1	1542	513	54.32	9.70	12
*TaSTP19*	TraesCS2B02G232900.1	1581	526	57.52	9.18	11
*TaSTP20*	TraesCS2B02G282400.1	1554	517	56.30	9.51	11
*TaSTP21*	TraesCS2B02G338400.1	1533	510	54.60	9.51	11
*TaSTP22*	TraesCS2B02G338500.1	1545	514	55.52	9.58	11
*TaSTP23*	TraesCS2B02G338600.1	1374	457	50.18	10.02	11
*TaSTP24*	TraesCS2B02G338700.1	1626	541	58.11	9.60	13
*TaSTP25*	TraesCS2B02G338700.2	1524	507	54.26	9.62	11
*TaSTP26*	TraesCS2B02G339100.1	1542	513	54.22	9.83	12
*TaSTP27*	TraesCS2D02G264200.1	1554	517	56.29	9.57	11
*TaSTP28*	TraesCS2D02G318200.1	1533	510	54.48	9.61	11
*TaSTP29*	TraesCS2D02G318300.1	1545	514	55.61	9.58	11
*TaSTP30*	TraesCS2D02G318400.1	1548	515	56.33	9.68	11
*TaSTP31*	TraesCS2D02G318500.1	1524	507	54.31	9.67	11
*TaSTP32*	TraesCS2D02G319100.1	1542	513	54.23	9.75	12
*TaSTP33*	TraesCS3A02G102800.1	1533	510	54.62	9.37	11
*TaSTP34*	TraesCS3A02G475200.1	1527	508	55.67	9.59	11
*TaSTP35*	TraesCS3B02G120300.1	1386	461	49.82	9.36	10
*TaSTP36*	TraesCS3B02G518800.1	1527	508	55.63	9.54	11
*TaSTP37*	TraesCS3B02G518900.1	1545	514	55.83	9.41	11
*TaSTP38*	TraesCS3D02G105000.1	1539	512	54.79	9.18	11
*TaSTP39*	TraesCS3D02G470200.1	1545	514	55.83	9.41	11
*TaSTP40*	TraesCS3D02G470300.1	1527	508	55.36	9.39	11
*TaSTP41*	TraesCS4A02G066200.1	1548	515	56.90	8.96	11
*TaSTP42*	TraesCS4A02G314900.1	1545	514	55.93	9.28	12
*TaSTP43*	TraesCS4A02G384900.1.cds1	1542	513	54.28	9.84	12
*TaSTP44*	TraesCS4B02G243500.1	1545	514	56.69	8.95	11
*TaSTP45*	TraesCS4B02G346400.1	1554	517	55.01	9.92	10
*TaSTP46*	TraesCS4B02G371700.1	1575	524	57.00	8.99	11
*TaSTP47*	TraesCS4B02G371700.2	1848	615	67.04	8.59	11
*TaSTP48*	TraesCS4D02G243100.1	1545	514	56.70	8.95	11
*TaSTP49*	TraesCS4D02G341400.1	1548	515	54.87	10.05	10
*TaSTP50*	TraesCS4D02G365800.1	1581	526	57.24	9.06	11
*TaSTP51*	TraesCS5A02G001100.1	1551	516	56.28	9.33	11
*TaSTP52*	TraesCS5A02G182600.1	1590	529	57.47	9.07	12
*TaSTP53*	TraesCS5A02G226500.1	1566	521	56.55	9.26	11
*TaSTP54*	TraesCS5A02G226700.1	1563	520	56.30	9.06	12
*TaSTP55*	TraesCS5A02G226800.1	1587	528	57.29	8.79	12
*TaSTP56*	TraesCS5A02G515200.1	1548	515	54.99	9.98	10
*TaSTP57*	TraesCS5A02G542600.1	1158	385	41.27	9.79	8
*TaSTP58*	TraesCS5A02G542600.2	1581	526	57.26	8.98	11
*TaSTP59*	TraesCS5B02G001000.1	1554	517	56.05	9.16	11
*TaSTP60*	TraesCS5B02G180800.1	1587	528	57.43	8.86	10
*TaSTP61*	TraesCS5B02G225100.1	1563	520	56.39	9.34	11
*TaSTP62*	TraesCS5B02G225300.1	1542	513	55.76	9.05	10
*TaSTP63*	TraesCS5B02G225400.1	1587	528	57.27	8.78	12
*TaSTP64*	TraesCS5D02G001600.1	1554	517	56.21	9.27	11
*TaSTP65*	TraesCS5D02G187100.1	1590	529	57.45	8.97	10
*TaSTP66*	TraesCS5D02G233900.1	1563	520	56.47	9.26	11
*TaSTP67*	TraesCS5D02G234100.1	1563	520	56.44	9.23	12
*TaSTP68*	TraesCS5D02G234200.1	1587	528	57.31	8.90	12
*TaSTP69*	TraesCS5D02G549900.1	1545	514	55.95	9.20	12
*TaSTP70*	TraesCS5D02G558100.1	1515	504	55.16	9.62	11
*TaSTP71*	TraesCS6A02G134000.1	1572	523	57.27	8.57	12
*TaSTP72*	TraesCS6A02G231700.1	1530	509	55.08	8.90	12
*TaSTP73*	TraesCS6A02G351600.1	1080	359	39.55	10.05	6
*TaSTP74*	TraesCS6B02G162200.1	1572	523	57.41	8.43	11
*TaSTP75*	TraesCS6B02G384700.1	1578	525	58.34	8.81	11
*TaSTP76*	TraesCS6D02G123300.1	1572	523	57.27	8.42	12
*TaSTP77*	TraesCS7A02G085900.1.cds1	1545	514	54.45	9.89	10
*TaSTP78*	TraesCS7A02G288800.1	1536	511	55.38	8.44	12
*TaSTP79*	TraesCS7B02G185800.1	1536	511	55.33	8.59	12
*TaSTP80*	TraesCS7D02G080200.1.cds1	1539	512	54.12	9.92	12
*TaSTP81*	TraesCS7D02G286600.1	1536	511	55.37	8.64	12

**Notes.**

a Length of the amino acid sequence

b Molecular weight of the amino acid sequence

c Isoelectric point of the TaSTP

d Number of transmembrane domains, as predicted by the TMHMM server

## Results

### Identification of the STP gene family in wheat

To identify the STP family genes in wheat, the Sugar_tr HMM profile (Pfam: PF00083) was used as a query in a BlastP search against the wheat genome database (International Wheat Genome Sequencing Consortium: IWGSC). A total of 476 candidate TaSTP proteins were identified from the wheat genome with an *E*-value of <1e^−5^. To identify the members of the TaSTP protein family, the proteins were checked for the presence of the Sugar_tr domain by the Pfam database (Finn et al., 2010) and the SMART database (Letunic & Bork, 2018). A total of 86 candidate STP proteins were discarded in this study because of a lack of a part of STP domain or complete STP domain. Finally, a total of 390 putative *TaSTP* genes were identified in the wheat genome through a HMMER analysis. A phylogenetic tree was constructed according to the protein sequences of 390 putative TaSTP and 14 AtSTP. The evolutionary tree was divided into three subgroups, and only the second subpopulation contains all *Arabidopsis* sugar transporters proteins, named AtSTP1 to AtSTP14 (Fig. 1, Group 2). These are all monosaccharide transporters and our focus was on the analysis of these monosaccharide transporters in wheat. There were 81 genes in wheat in the same group as in *Arabidopsis thaliana*. According to the position of the genes on the chromosomes, the 81 *TaSTP* genes were unevenly located on the wheat chromosomes and thus were named *TaSTP 1* to *TaSTP 81*. The general information on the 81 TaSTP members, including molecular weight, isoelectric point, CDS length, amino acid number, and transmembrane domain information, is summarized in Table 1. These genes were distributed on either the long or short arm of the A, B, and D chromosomes. Among them, chromosomes 1A, 2A, 3A, 4A, 5A, 6A and 7A had four, seven, two, three, eight, three, and two *TaSTPs*, respectively; chromosomes 1B to 7B had four, eight, three, four, five, two, and one TaSTPs, respectively; and chromosomes 1D to 7D had three, six, three, three, seven, one, and two *TaSTPs*, respectively (Table 1). We found that chromosomes A, B, and D had 29, 27, and 25 *TaSTP* genes, respectively. The open reading frames (ORFs) and the protein lengths of the *TaSTP* genes ranged from 1,080 to 1,848 bp in length and 359 to 615 amino acids in length, respectively (Table 1). The predicted molecular weights of the *TaSTPs* ranged from 39.55 kDa to 67.04 kDa. The theoretical isoelectric points (pIs) of the *TaSTP* proteins ranged from 8.42 to 10.05. Moreover, 77 of the 81 TaSTPs contained 10 to 12 conserved transmembrane domains (TMDs), and most had 11 or 12; however, *TaSTP73*, *TaSTP57*, and *TaSTP6* carried only six, eight, and nine TMDs, and TaSTP24 carried 13 TMDs (Table 1).

### Sequence structure features of the *TaSTP* s

**Figure 2:**
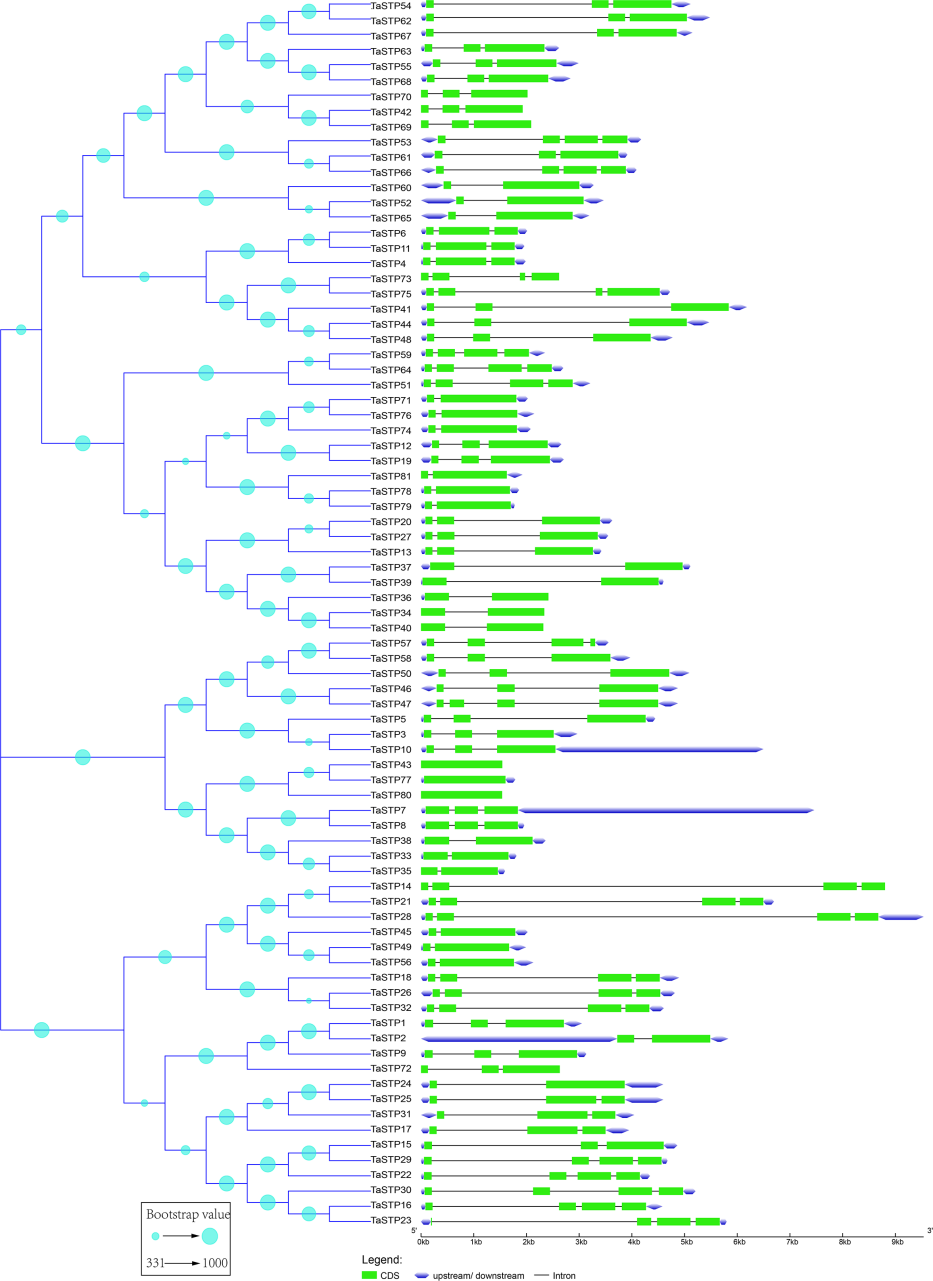
Predicted TaSTP protein phylogeny and exon-intron structure. The exon–intron structures of the 81 TaSTP genes were generated by comparing the coding sequences and the corresponding genomic sequences using the GSDS website (http://gsds.gao-lab.org/) and optimized using TBtools. The construction method of the phylogenetic tree is the same as that of Fig. 1.

According to the neighbour-joining phylogenetic tree of the *TaSTP*s gene sequences, which was constructed using 1,000 bootstrap replicates by MEGA v7.0 (Pennsylvania State University, Philadelphia, PA, USA), the 81 *TaSTP* genes could be classified into three distinct groups according to the similarity of their gene sequences, and the bootstrap value ranged from 331 to 1,000 (Fig. 2). The largest group contained 42 *TaSTP* genes, and the other two groups contained 16 and 23 *TaSTP* genes, respectively. The *TaSTP* gene structures commonly had two, three, or four CDS divided by one, two, or three introns, except for *TaSTP43*, *TaSTP77,* and *TaSTP80* with one CDS. Furthermore, most of the TaSTP genes that were classified into the same subgroup exhibited similar gene structures, such as *TaSTP42*,*69* and *TaSTP70*, *TaSTP6*,*11* and *TaSTP4*, *TaSTP71*,*76*, and *TaSTP74* (Fig. 2)*.*

**Figure 3:**
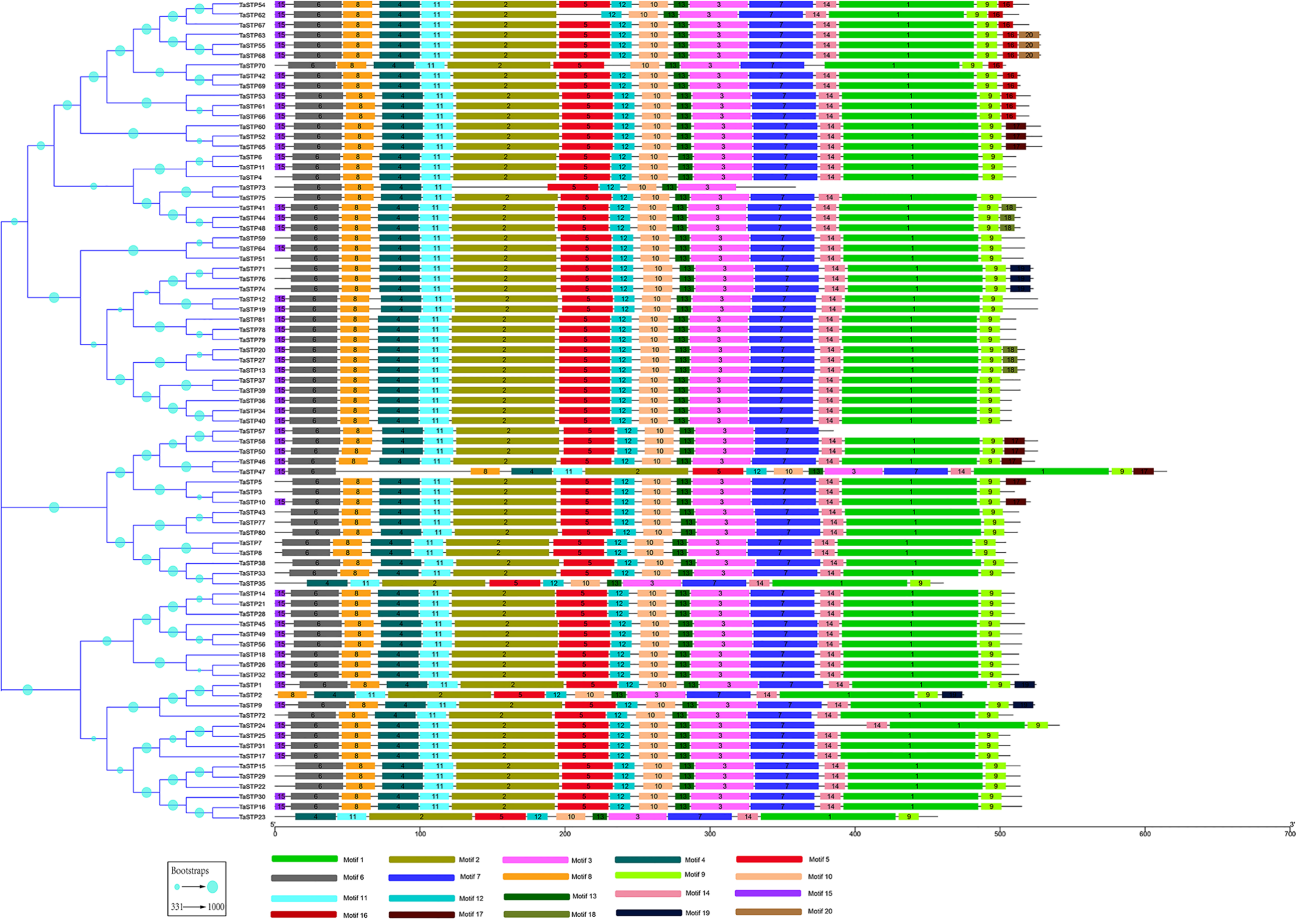
MEME motif search results of the predicted TaSTP proteins by MEME suite 5.0.1. The non-conserved sequences are shown by black lines, and the different motifs are represented by different coloured boxes numbered at the centre of the box and bottom of the figure. In addition, the lengths of the motifs in each protein are proportional. The phylogenetic tree is the same as that in Fig. 2.

**Figure 4:**
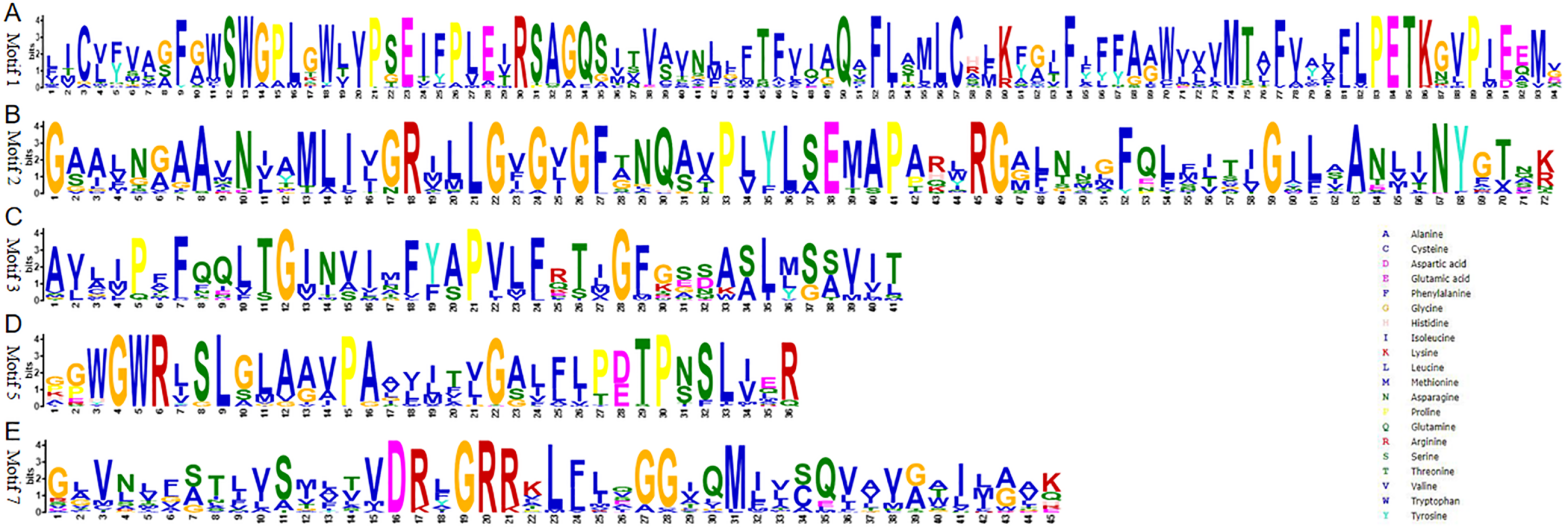
Conserved motifs of Sugar_tr in STP proteins. The motif logos and the amino acid compositions of the Sugar_tr motifs are as follows: Motif 1, Motif 2, Motif 3, Motif 5 and Motif 7. The *x*-axis represents the amino acid type and position. The *y*-axis shows the overall height of the amino acid stacks, which indicates the sequence conservation at a given position, while the height of the individual symbols within a stack indicates the relative frequency of a nucleotide base at that position.

To understand their functional regions, the conserved motifs of the TaSTP proteins were identified through the MEME website. Twenty motifs with minimum and maximum motif width set from six to 200 in the 81 proteins were analysed using the MEME tool. The results from MEME agreed with those from the phylogenetic tree (Fig. 3). It was found that most of the TaSTP proteins contained 15 or 16 motifs. *TaSTP2,15,22* and *29* had 14 motifs without motif 15 or another motif; *TaSTP23* and *TaSTP35* had 12 motifs without motifs six, eight, and 15; and *TaSTP73* had only nine motifs without motifs one, two, seven, nine, 14, and 15 (Fig. 3). We used Pfam to further analyze the 20 motifs and found that motifs one, two, three, five, and seven contained the Sugar_tr region. These five motif widths were 94, 72, 41, 36 and 45, respectively (Fig. 4, Table S2).

### Phylogenetic analysis of the TaSTP genes in seven species of monocotyledons and dicotyledons

**Figure 5:**
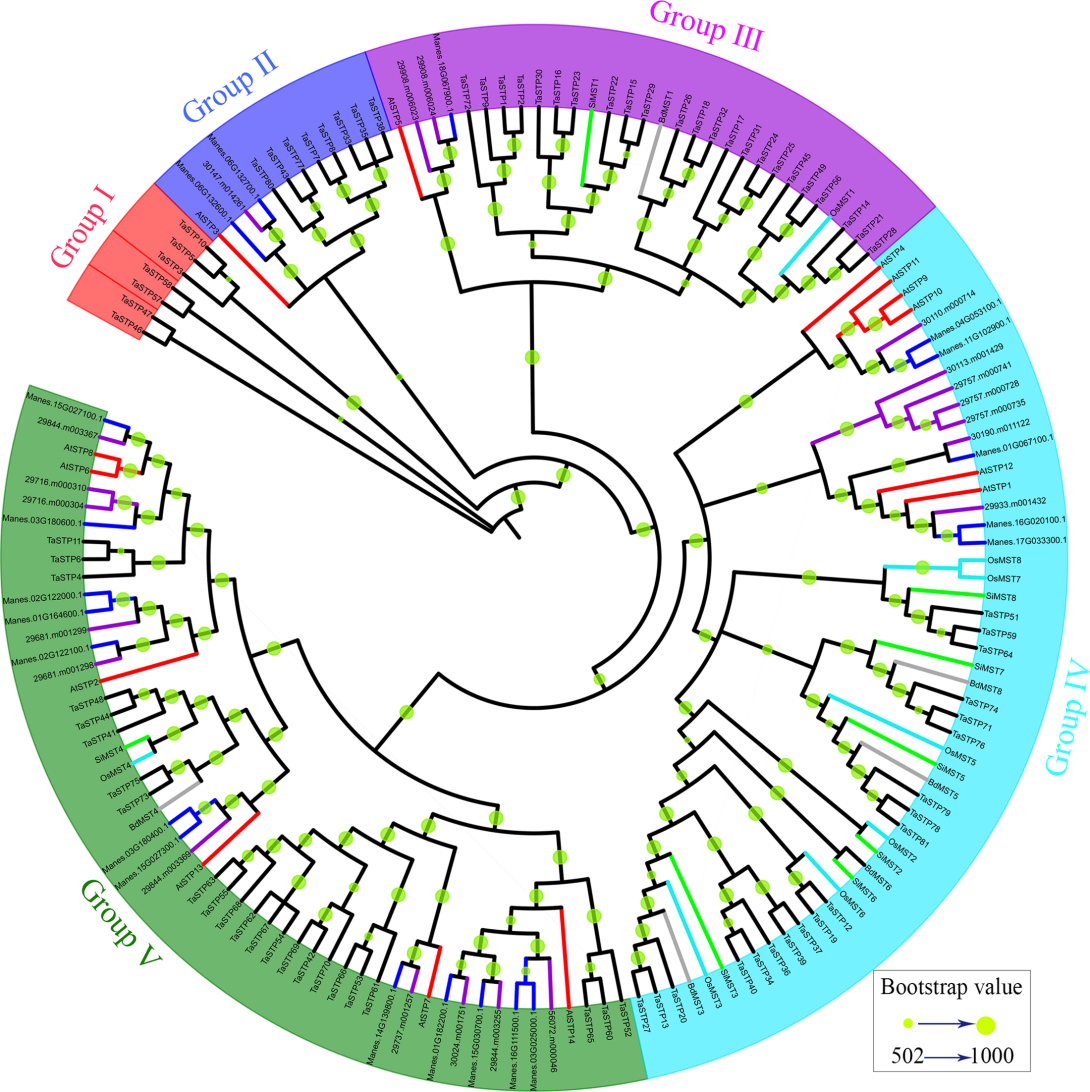
Phylogenetic relationship of the TaSTP proteins in seven plant species. The evolutionary relationship is presented using a phylogenetic tree. Five groups, which are named Group I to Group V are marked with diffirent lines. The bootstrap values of 502 to 1,000 are represented by the size of the green circles.

To investigate the evolutionary relationships of the TaSTP gene family in monocotyledons of graminaceae (wheat, foxtail millet, brachypodium distachyon, and rice) and dicotyledons such as *Arabidopsis*, cassava, and *Ricinus communis*, we used the STP genes from wheat (81), brachypodium distachyon (6), foxtail millet (8), rice (8), cassava (20), *Ricinus communis* (20), and *Arabidopsis* (14) to construct a N-J phylogenetic tree usng MEGA 7.0 software with 1,000 bootstraps (Table S3). In the phylogenetic tree, we use different-colored clades to represent different species: wheat (black), brachypodium distachyon (gray), foxtail millet (green), rice (azure), *Arabidopsis* (red), cassava (blue), and Ricinus communis (purple). The results of the phylogenetic tree indicated that it can be roughly divided into five groups: Group I was one small branch with seven cloned wheat STP genes (*TaSTP3,5,10,46,47,57,58*), and the other large branch; Group II to Group V mainly contained the other genes, characterized by blue, purple, sky blue, and green areas, respectively. The branches of Group II in the phylogenetic tree show that eight STP genes (*TaSTP7, 8, 33, 35, 38, 43, 77, 80*) and AtSTP3 were brought together. Group III consisted of twenty-three TaSTPs (*TaSTP1*, 2, 9, 14-18, 21-26, 28-32, 45, 49, 56, and 72), two RcSTPs, one MeSTP, BdMST1, AtSTP3 and SiMST1, and OsMST1 (Fig. 5). Nineteen TaSTPs (TaSTP*12, 13, 19, 20, 27, 34, 36, 37, 39, 40, 51, 59, 64, 71, 74, 76, 78, 79,* and *81*) clustered with six AtSTPs (AtSTP*1, 4, 9-12*), seven RcSTPs, five MeSTPs, four BdMSTs, and six SiMSTs in Group IV (Fig. 5). Group V consisted of twenty-three TaSTPs (TaSTP*4, 6, 9, 11, 41, 42, 44, 48, 52-55, 60-63, 65, 66-70, 73,* and *75*), six AtSTPs (AtSTP*2, 6, 7, 8, 13,* and *14*), twelve MeSTPs, ten RcSTPs, BdMST*4*, SiMST*4*, and OsMST*4* (Fig. 5).

### Chromosome location and gene duplication analysis

**Figure 6:**
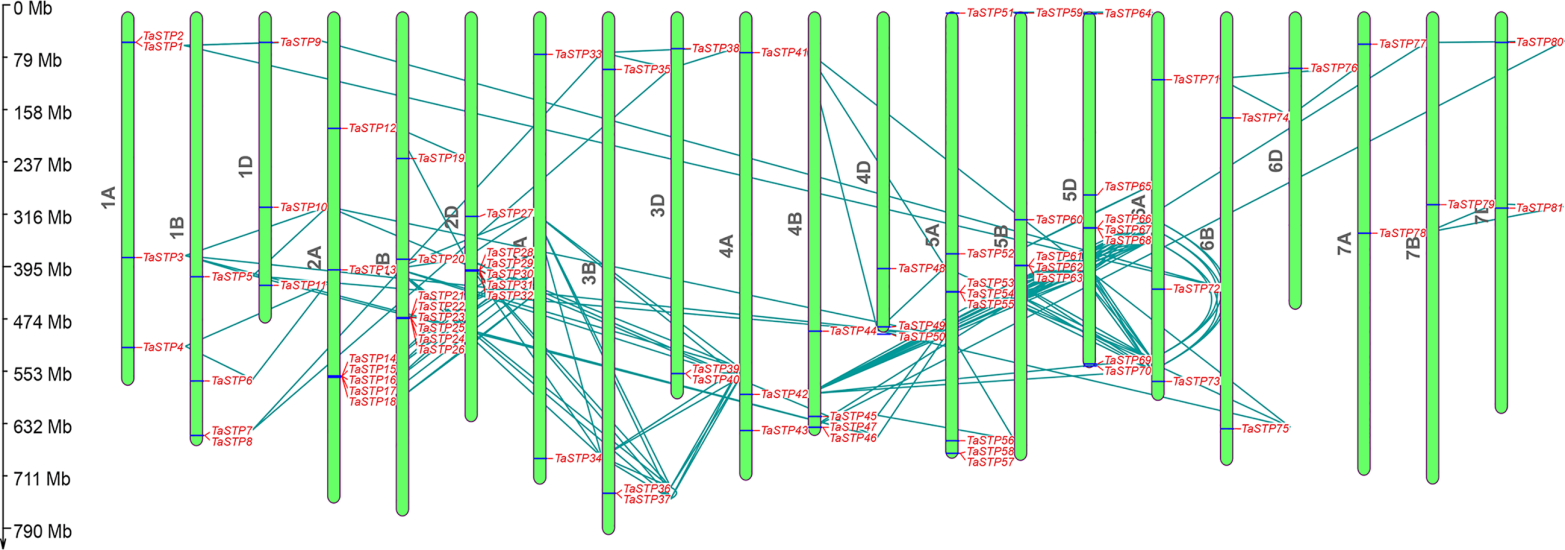
Chromosomal locations of the wheat sugar transporter (TaSTP) genes. Distribution of the TaSTP genes on the wheat chromosomes according to the linkage map. Tandem duplicates are connected by dark cyan coloured lines. In total, 81 TaSTP genes were mapped to 21 chromosomes (1A-7A, 1B-7B and 1D-7D). The scale is in bp (base pair). A total of six gene clusters containing three or more than three genes were distributed on the 2A, 2B, 2D, 5A, 5B, and 5D chromosomes, respectively.

**Figure 7:**
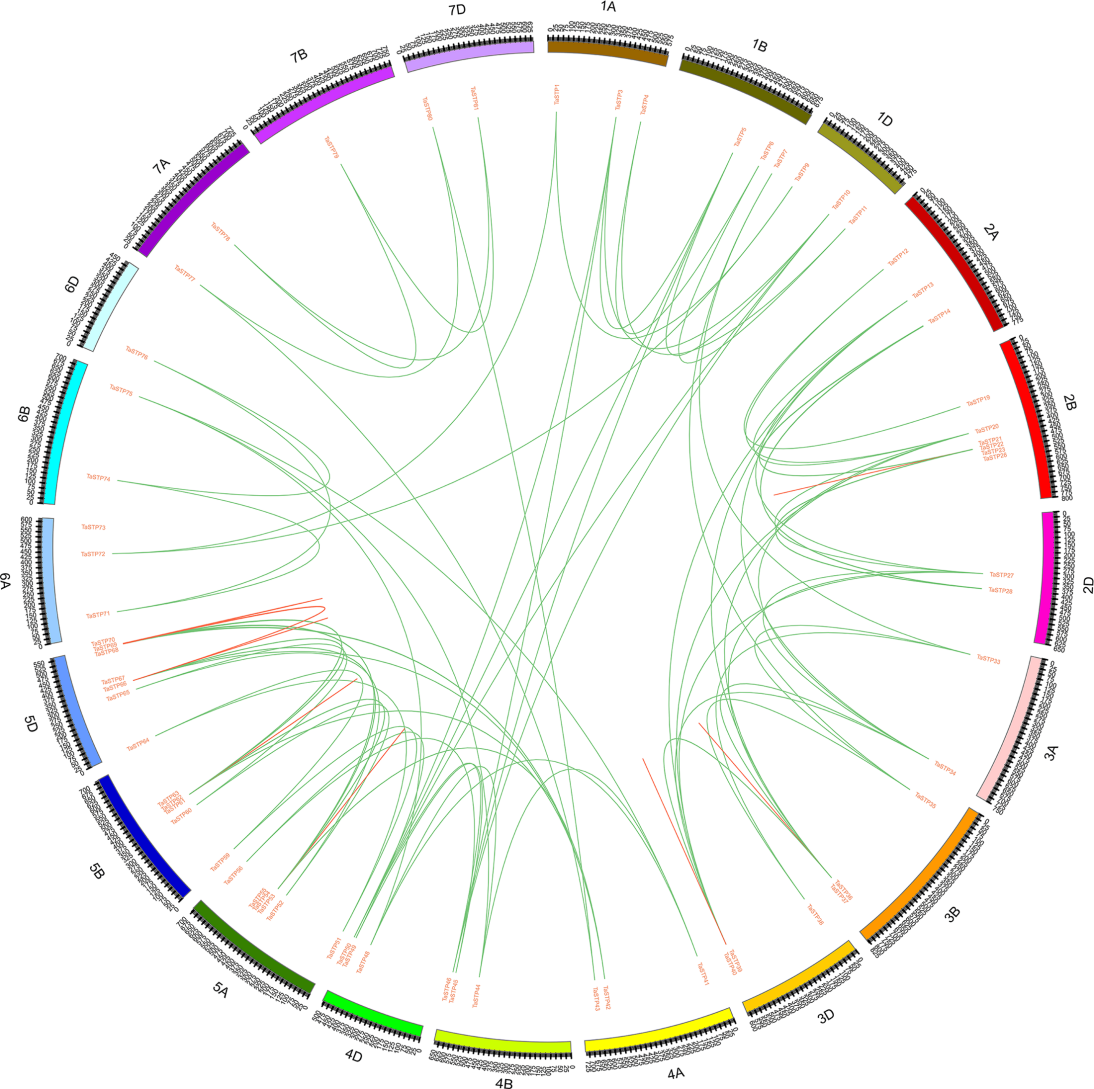
Localization and synteny of the TaSTP genes in the wheat genome. The TaSTP genes in wheat (TaSTP) were mapped to different chromosomes. The chromosome number is indicated on the outside. The numbers along the chromosome boxes represent sequence lengths in megabases. Gene pairs with a syntenic relationship are joined by a green line. The duplicated genes are joined by a red line.

The approximate locations of the TaSTP genes on the chromosomes of wheat were determined by MapDraw software according to http://plants.ensembl.org/Triticum_aestivum/Info/Index. We found that the 81 genes were located separately on chromosomes A, B, and D. Moreover, *TaSTP14-TaSTP18*, *TaSTP21*-*TaSTP26*, *TaSTP28-TaSTP32*, *TaSTP53-TaSTP55*, *TaSTP61*-*TaSTP63*, and *TaSTP66*-*TaSTP68* formed a gene cluster with more than three genes on chromosomes 2A, 2B, 2D, 5A, 5B, and 5D, respectively (Fig. 6). We also investigated the tandem duplications and whole-genome duplications of the 81 TaSTP genes. As shown in Fig. 6, the TaSTP genes were differentially distributed on 21 wheat chromosomes. Among these TaSTP genes, 159 pairs of genes exhibited whole-genome duplications/segmental duplications (Table S4). A total of 22, 23, and 21 TaSTP genes were distributed in the A, B and D sub-genome, respectively (B¿A¿D) as shown with the tandem replication events of multiple genes in Fig. 7. Initial gene loss likely occurred in the A genomes following tetraploidy and D genomes following hexaploidy to decrease functional redundancy and define the core wheat genes. There were nine, eleven, eight, nine, eighteen, six, and give genes in groups one through seven of the chromosomes, which showed three obvious gradients between groups two and five, and one, three, four and the other two groups. Specifically, chromosomes 5A, 5B and 5D had six, five, and seven genes, respectively, whereas chromosome 7B had only one TaSTP gene. These results indicated that the distribution of the TaSTP genes was not random in chromosomes and that gene replication events might have occurred in chromosomes two and five and may involve gene functions.

**Figure 8:**
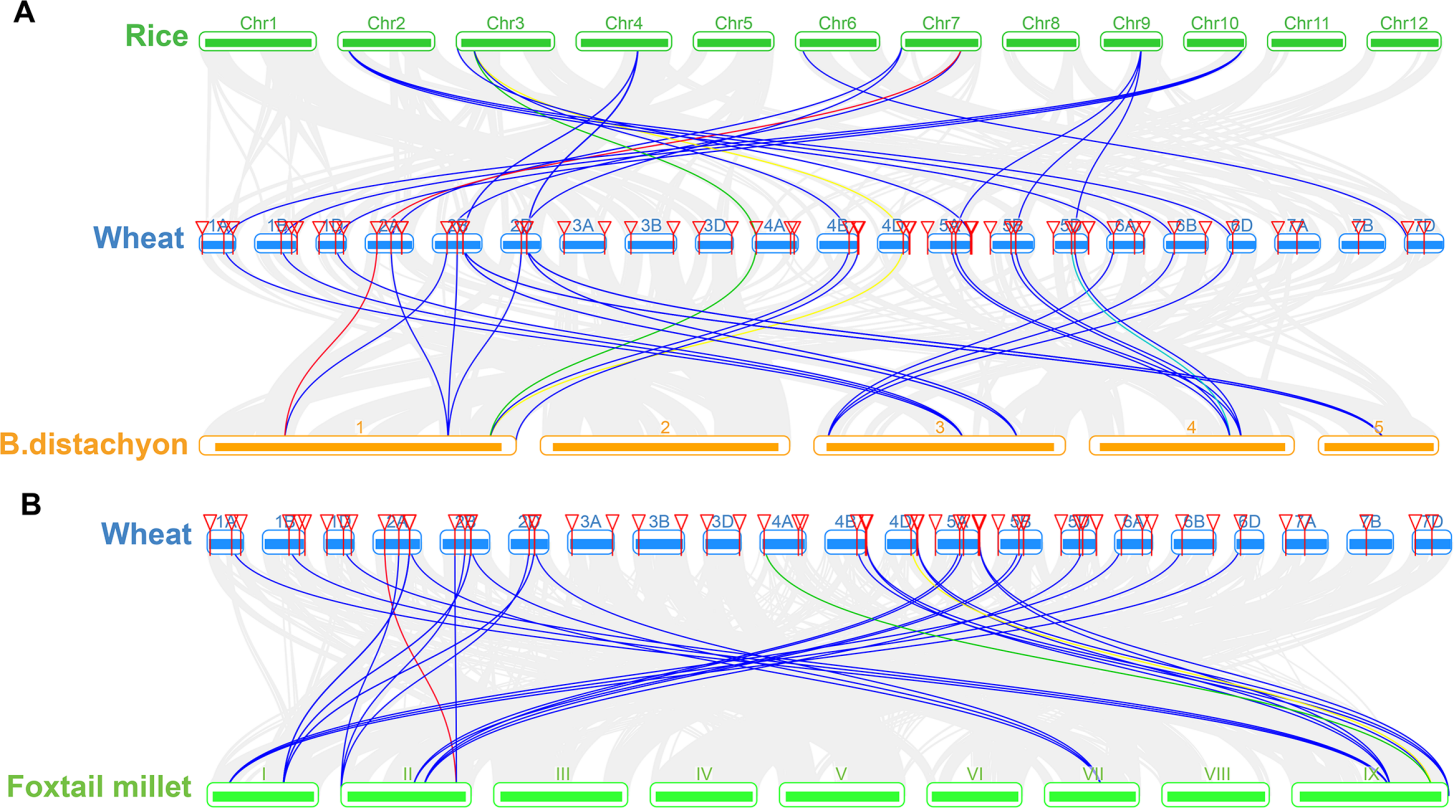
Synteny analysis of TaSTP genes between wheat and three representative plant species. Gray lines in the background indicate the collinear blocks within wheat and other plant genomes, while the other color lines highlight the syntenic TaSTP gene pairs. The specie names ‘Wheat’, ‘Rice’, ‘B. distachyon’, and ‘Foxtail millet’ indicate *Triticum aestivum*, *Oryza sativa*, *Brachypodium distachyum* and *Setaria italica*, respectively. (A) Synteny analysis of TaSTP genes between wheat and rice and *B. distachyon*; (B) Synteny analysis of TaSTP genes between wheat and foxtail millet.

We constructed three comparative syntenic maps of wheat associated with three representative species, including rice, foxtail millet, and brachypodium distachyum to further infer the phylogenetic mechanisms of the wheat TaSTP family. A total of 25 orthologous TaSTP gene pairs were found between wheat and rice and brachypodium distachyum (Table S5; Fig. 8A). These TaSTP genes were found to be associated with at least three syntenic gene pairs (particularly between rice and brachypodium distachyum genes), such as *TaSTP12*, *TaSTP4,1* and *TaSTP48* (shown as different color lines in the figure). These genes may have played an important role in the STP gene family during evolution. Moreover, 38 orthologous TaSTP gene pairs were found between wheat and foxtail millet (Table S5; Fig. 8B). We calculated the Ka/Ks ratios of the STP gene pairs to better understand the evolutionary constraints acting on STP gene family. All orthologous STP gene pairs had Ka/Ks < 1, suggesting that the wheat STP gene family might have experienced strong purifying selective pressure during evolution (Table S6). These results suggested that the STP genes of monocot have strong relationships.

### Strong purifying selection for the TaSTP gene pairs in wheat

**Figure 9:**
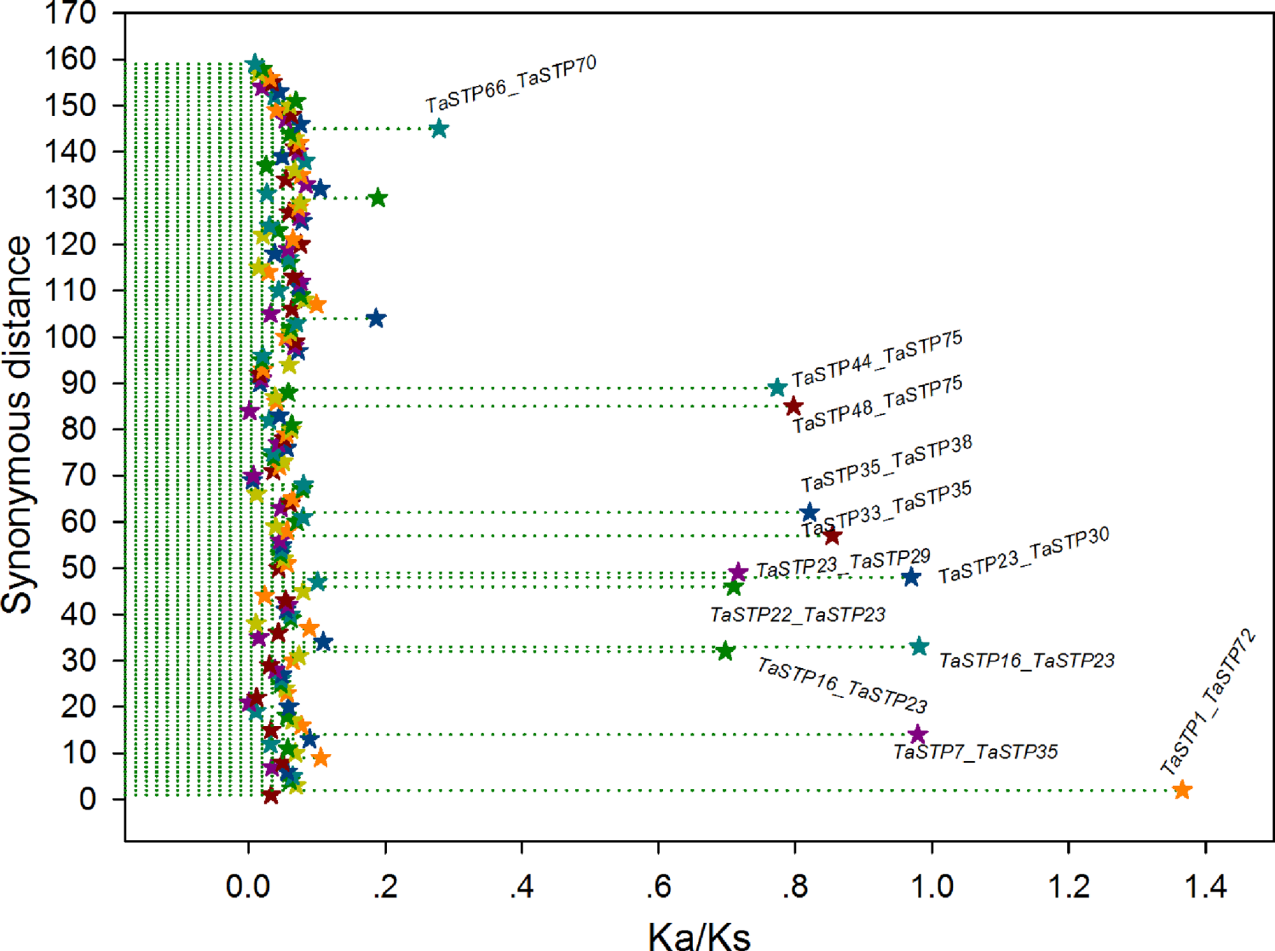
The Ka/Ks ratios of the gene pairs in the duplication blocks. The *x*- and *y*-axes denote the Ka/Ks ratio and the synonymous distance for each gene pair, respectively. The Ka/Ks values are presented by the star symbol.

In general, a nonsynonymous (Ka)/synonymous (Ks) ratio > 1 indicates positive selection, Ka/Ks ¡ 1 indicates purifying selection with functional constraints, and Ka/Ks = 1 indicates neutral selection. In our study, the Ka/Ks ratios of most of the TaSTP paralogous pairs were less than 1, and the ratio of only 1 paralogous pair was more than 1, namely, *TaSTP1 & TaSTP72*. Remarkably, of these Ka/Ks ratios <1, 147 TaSTP paralogous pairs were less than 0.27 and appeared to be under strong purifying selection, 11 were between 0.27 and 0.98 and appeared to be under purifying selection, and one appeared to be under positive selection (Fig. 9, Table S6).

### Expression profiles of the TaSTP genes

The RNA-seq data from http://www.wheat-expression.com/ were used to explore the expression profiles of the TaSTP genes in different tissues and under different abiotic stresses. According to the heat map results, the expression level of 76 TaSTP genes (genes with the same transcript were deleted) could be classified into A (yellow), B (blue), and C (pale red) pattern groups (Fig. S1). In the pattern group A (yellow), most genes in the different tissues showed low or no expression, and the genes *TaSTP28*, *TaSTP21*, *TaSTP1*, *TaSTP68*, *TaSTP55,* and *TaSTP63* were expressed in the roots of Chinese spring wheat. In the B pattern group (light blue), no gene expression was detected. Conversely, in the C pattern group (pale red), most genes, such as *TaSTP27* and *TaSTP48,* in the different tissues showed positively-regulated expression and, specifically, a higher expression level in the flag leaf blades and roots. Some genes were highly expressed in the seed coat, such as *TaSTP52*, *TaSTP53*, *TaSTP60, TaSTP61, TaSTP65,* and *TaSTP6 6*. In the stressed seedling leaves, the expression pattern was roughly divided into two groups: one group showed negative regulation with low expression compared with that of the control, such as the expression patterns in heat, drought stress or heat and drought combined stress at one hour and six hours, and the other group was positively or negatively regulated by PEG6000 stress at two hours and 12 h in nine-day-old seedlings of Giza168 and Gemmiza 10. Furthermore, compared with the gene expression in the no stress control, most genes were upregulated in shoots under cold stress at 4 °C for two weeks, except for the genes *TaSTP44, TaSTP* 53, *TaSTP56,* and *TaSTP61* (Fig. S1).

### Bioinformatic-based protein interaction network analysis of the TaSTPs using STRING 10.5 and GO analyses

To explore the relationship between all the identified TaSTPs, we created a protein interaction network by searching the 81 proteins against the TAIR (The *Arabidopsis* Information Resource) database in STRING. We found 17 proteins with hits in the STRING database. These proteins interactions were divided into three groups: the first group (blue) was transglycosidase (DIN10), which is involved in the synthesis of raffinose, a major soluble carbohydrate in seeds, roots and tubers, and the second group (green) was trehalose phosphate synthase and trehalose-6-phosphate phosphatase (which removes the phosphate from trehalose 6-phosphate to produce free trehalose that accumulates in plants and may improve abiotic stress tolerance). The third group (red) was beta-fructofuranosidase (ATBFRUCT1, that can use sucrose and 1-kestose), glutamate synthase, and glutamate dehydrogenase, which are required during photorespiration (Fig. S2).

**Figure 10:**
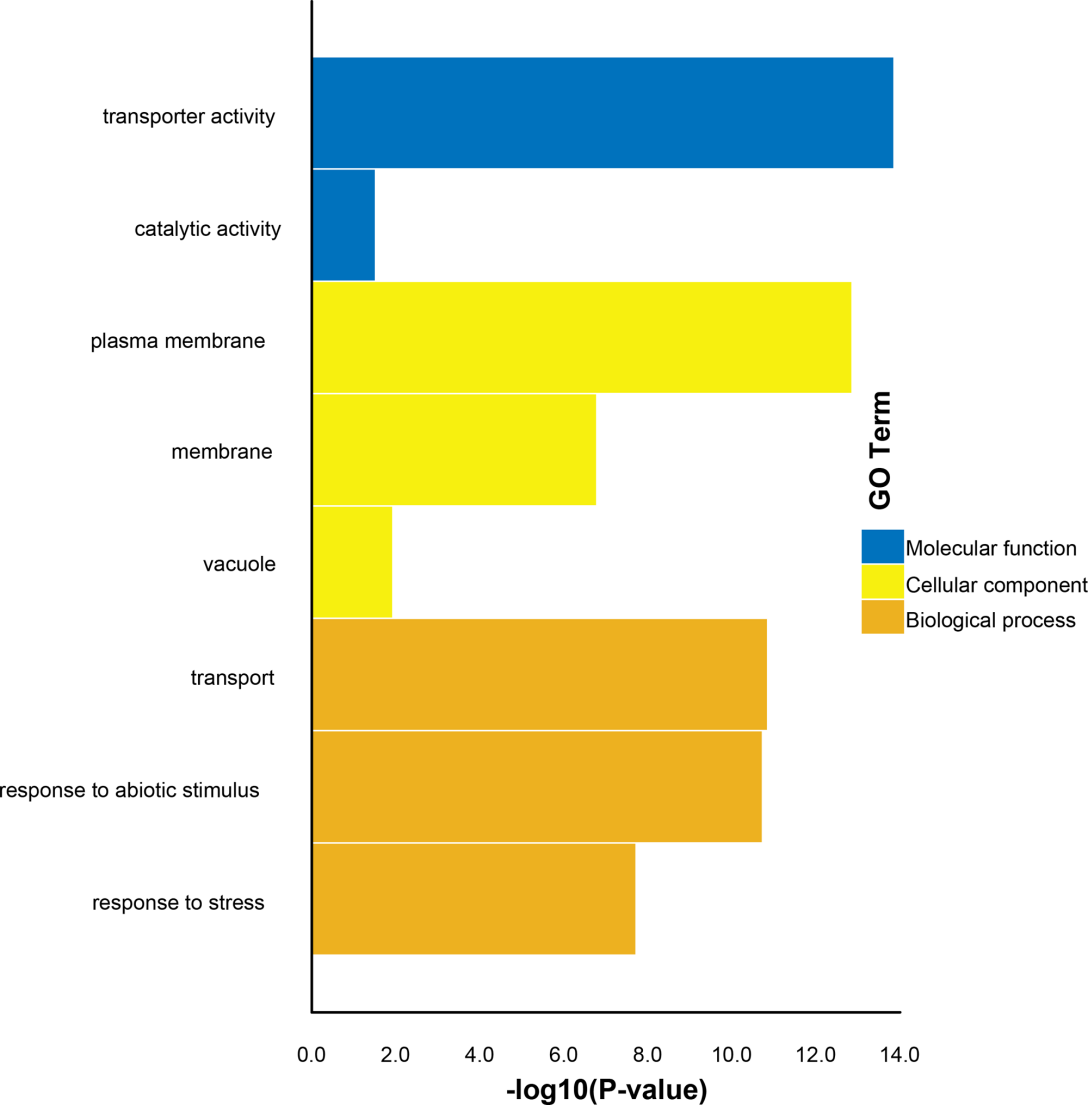
Enrichment of GO terms in the three main categories for the 81 TaSTP genes considered in this study. Three main categories, namely, molecular function, cellular component and biological process, are shown in blue terms, bright yellow terms, and brownish yellow terms, respectively.

To further identify the functions of the putative TaSTP proteins in wheat, molecular functions, cellular components, and biological pathway categories were predicted using GO annotation analysis. Based on amino acid similarity, the results of the GO analysis showed that 81 TaSTP proteins were categorized into eight functional categories (Fig. 10). Among the molecular functions, transporter activity and catalytic activity were predominant. The analysis of the cellular component annotations revealed that most of the TaSTP proteins were predominantly localized to the plasma membrane, membrane and vacuole. Furthermore, 81 TaSTP proteins were assigned to transport, response to abiotic stimulus, and response to stress based on biological process analyses.

### Phenotype comparison of seedling roots, stems, and leaves under stress

Stress treatment was performed when seedlings grew to the second-true leaf stage. Wheat seedlings were divided into four groups with 100 seedlings per group and were treated with 150 mmol L^−1^NaCl, 10% PEG6000 solution, 10% PEG6000 solution+150 mmol L^−1^NaCl, or the control. Investigations were carried out after three, six, and nine days of stress, and the length and width of the roots, stems and leaves were accurately measured using vernier callipers. The phenotypes of the seedling roots, stems, and leaves under stress were different from those of the normal seedlings, and the most obvious difference was in root length (Figs. S3A–S3L). Specifically, at the three time points of stress, the root length of control was slightly elongated, while NaCl stress and PEG6000+NaCl stress did not change significantly in the root length. Interestingly, the root lengths of plants under PEG6000 stress continued to increase and were significantly different from that of the control on the sixth day and ninth day of stress (*P* < 0.01) (Fig. S4A). In contrast, the root width of the control, NaCl stress, and PEG6000+NaCl stress plants gradually decreased as the plants grew. The root width of PEG6000 tended to be thinner on the sixth day and then thicker on the ninth day, but it was significantly thinner than that of the control (Fig. S4B). In the stems, there was no significant change in length among the four types of seedlings, but the diameter of the seedlings treated with stress was thinner than that of the control on the sixth and ninth days of treatment and showed significant (*P* < 0.05) or extremely significant (*P* < 0.01) differences. (Figs. S4C and S4D). The lengths of first true leaves of control were slightly shorter than those of the stressed leaves, but the width was wider than that of the stressed leaves (Figs. S4E and S4F). The length and width of the second true leaves of the NaCl-stressed and PEG6000+NaCl-stressed seedlings were significantly shorter than those of the control leaves (*P* < 0.01). However, the PEG6000-stressed leaves had approximately the same length and width as the control leaves (Figs. S4G and S4H).

### Expression characteristics of wheat *TaSTP* genes using qRT-PCR analysis

**Figure 11:**
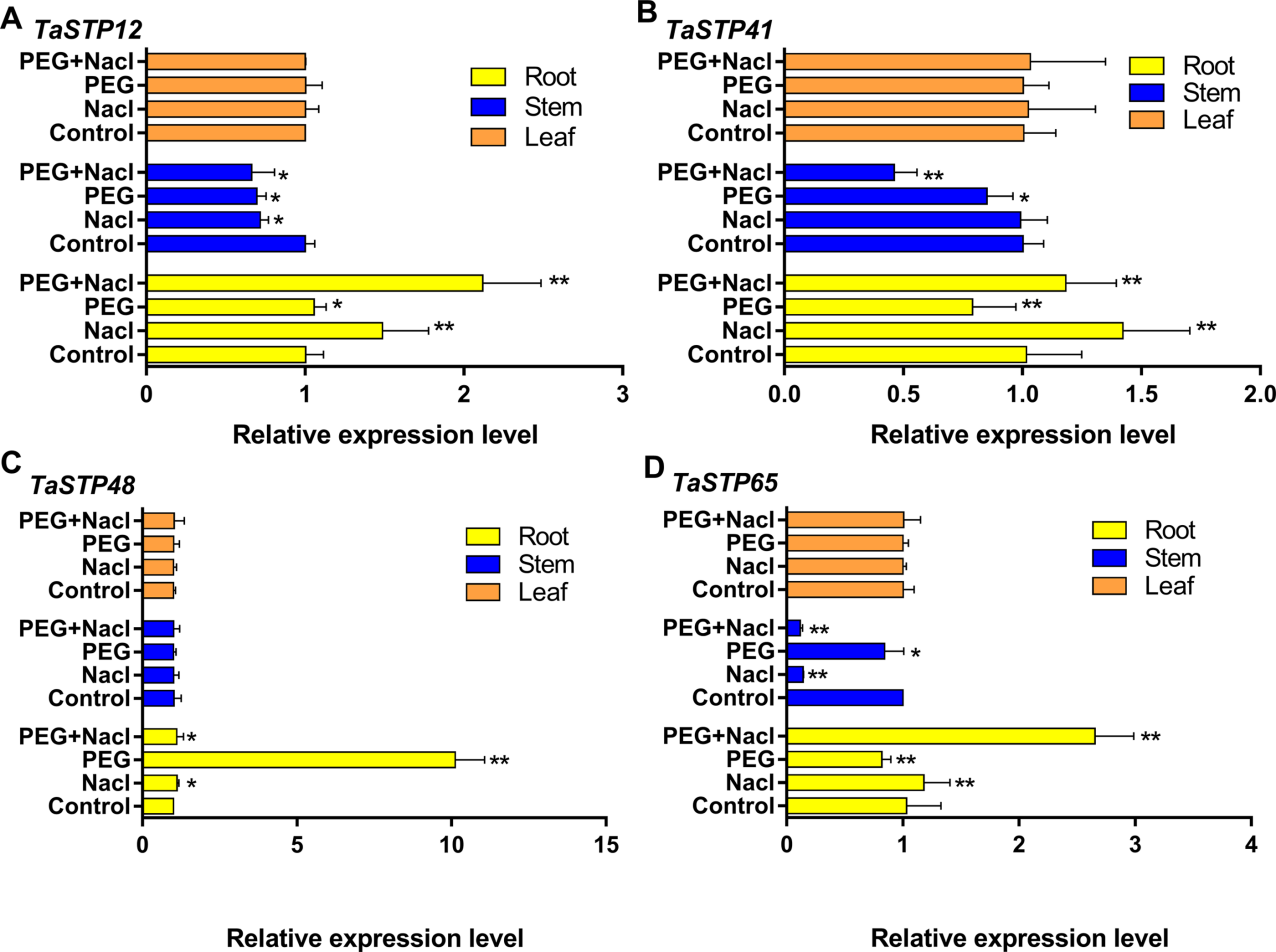
Comparison of the relative expression levels of four TaSTPs involved in different tissues on sixth day of the stress treatments. The *x*-axes indicate the relative gene expression levels; the *y*-axes indicate the different stress treatments in the root, stem and leaf tissues. A to D show the relative expression level of *TaSTP 12*, *TaSTP 41*, *TaSTP 48* and *TaSTP 65* genes in root, stem and leaf under NaCl stress, PEG6000 stress and their combined stresses and the control after 6 days, respectively. Capped lines indicate standard error. * *P* < 0.05; ** *P* < 0.01.

We investigated the expression levels of the *TaSTP* genes during stress at the sixth day of wheat development to further explore *TaSTP* gene functions in wheat. First, we randomly selected four genes, *TaSTP12, TaSTP41, TaSTP48,* and *TaSTP65*, for qRT-PCR analysis, and their expression is quantified in Fig. 11. The results showed that these genes were expressed in the roots, stems, and leaves. Interestingly, the expression levels of the individual genes in the leaves were not the same but the expression of the same genes in the leaves was not affected by stress. For the gene *TaSTP 12*, the expression levels in the stressed wheat stems were similar but they were lower than those in the control stems; the difference was significant (*P* < 0.05). The expression of *TaSTP 12* was significantly higher in the stressed roots than in the control roots, and the differences were extremely significant, with a 1.4-fold change and 2.11-fold change in the roots of the NaCl-stressed and PEG6000+NaCl-stressed roots, respectively (*P* < 0.01) (Fig. 11A). Compared with *TaSTP 41* expression in the control stems, *TaSTP 41* expression was significantly downregulated in the PEG6000+NaCl-stressed (2.17-fold change, *P* < 0.01) and PEG6000-stressed stems (1.18-fold change, *P* < 0.01), but the NaCl-stressed did not show a difference. *TaSTP 41* expression was significantly upregulated in the PEG6000+NaCl-stressed (1.16-fold change, *P* < 0.01) and NaCl-stressed (1.4-fold change, *P* < 0.01) roots relative to the control roots. However, the expression was significantly downregulated (1.26-fold change, *P* < 0.01) in the PEG6000-stressed roots relative to the control roots (Fig. 11B). A difference in expression was not detected for the gene *TaSTP 48* between the control stems and stressed stems. In the roots, the expression level of *TaSTP 48* increased in the stressed roots compared with that in the control roots, and this was especially the case with PEG6000 stress, which showed more than a 10.04-fold change (*P* < 0.01) (Fig. 11C). *TaSTP 65* expression was significantly downregulated in the PEG6000+NaCl-stressed (8.62-fold change, *P* < 0.01) and NaCl-stressed (7.07-fold change, *P* < 0.01) stems compared with that in the control stems. In the roots, significantly elevated expression levels were seen with sodium chloride stress, specifically, a 1.15-fold change and a 2.57-fold change with NaCl stress and PEG6000+NaCl stress, respectively, (*P* < 0.01). Moreover, *TaSTP 65* showed a lower expression with a 1.26-fold change (*P* < 0.01) in the PEG6000-stressed roots compared to that in the control roots (Fig. 11D).

## Discussion

A large number of studies have shown that plants suffering from drought or salt stress can significantly increase their soluble sugar content, and soluble sugar is mainly involved in osmotic adjustment within the cell. The soluble sugar content is significantly positively correlated with plant stress and can be used as a screening index for stress resistance (Gupta & Kaur, 2006; Rizhsky et al., 2004). Furthermore, recent studies have shown that glucose and sucrose are important regulators of plant growth and gene expression, with hormone-like primary messenger functions, and participate in the regulation of plant growth and development under biotic and abiotic stress (Rolland, Baena-Gonzalez & Sheen, 2006). The most important function of sugar transporters is their role in mediating the long-distance transport of sugars in the phloem during plant development. STP genes have been cloned from various plants and organs, such as *Arabidopsis* and sorghum, and food crops, including rice, barley, maize. However, 14 STP genes were found in *Arabidopsis*, which has a relatively small genome (125 Mb), so there are certainly many STP genes waiting to be discovered in the large wheat genome (17 Gb). We globally analysed the gene structure, protein motifs and phylogenetic tree of the TaSTP family in wheat for the first time. Furthermore, we investigated the expression of the TaSTP genes in different tissues during stress at sixth day of wheat development using qRT-PCR analysis to deeply explore TaSTP gene functions.

Eighty-one TaSTP genes were identified in the wheat genome through a BLAST search and HMMER analysis (Table 1), and they were distributed in the A, B, and D genomes. MEME results showed that most of the genes contained 12–13 motifs, and some were missing one or several motifs, indicating that the loss of the N-terminal or C-terminal regions may have occurred in some TaSTP members during evolution (Fig. 3). These results were in agreement with those obtained by studies in tomato, grape, and cassava (Liu et al., 2018b; Reuscher et al., 2014). The result of phylogenetic analysis showed that 81 TaSTPs, together with other STP proteins from wheat, *Brachypodium distachyon*, foxtail millet, rice, *Arabidopsis*, cassava, and *Ricinus communis* were classified into five groups and that the cloned wheat STP genes were in the same broad category as the TaSTP members we identified. Therefore, a close relationship between the cloned wheat STP proteins and TaSTP members indicated that these proteins possibly possess similar biochemical properties. Additionally, the RNA-seq data from seventeen tissues indicated that the TaSTP genes are differentially expressed in various tissues. Moreover, responses to abiotic stimuli and responses to stress were shown from the results of the biological process analysis of GO annotation. These results indicated that sucrose transporters are closely related to stress.

Thus, based on the phenotype comparisons, qRT-PCR was performed for the stressed wheat seedlings. Interestingly, the expression levels of the individual genes were different in the leaves, but the expression levels of the same genes were basically not affected by stress in the leaves (Fig. 11). Our findings also indicated that the expression of the sugar transporter gene in leaves may not be affected by short-term stress processes. The pattern of STP expression in the wheat stems subjected to stress differed from that in the leaves. The expression pattern of the TaSTP genes was downregulated in stressed plants compared with that in the control plants. Among them, *TaSTP12* was significantly downregulated under the three stress patterns. *TaSTP41* showed no significant downregulation under NaCl stress but showed significant downregulation under PEG-related stress. *TaSTP48* did not show significant differences, but there was slight trend for downregulation. *TaSTP65* showed significant downregulation in all stressed stems, and it showed an extremely significant downward trend under NaCl-related stress. These results are in agreement with those obtained from cold-stressed seedling stems from the RNA-seq data from the website (*TaSTP12 ,48,65*). These results may be related to the thin and short stems that grew under stress. Some genes have a clear propensity for stress response and the *TaSTP65* gene may be closely related to NaCl stress in the stems. It has been shown that STP expression does not always correspond to phloem unloading, and consistent patterns of STP expression under stress indicate which types of STPs are involved in stress tolerance (Truernit et al., 1996; Julius et al., 2017).

In our study, the most striking change was observed in STP genes in the roots and *STP 13* expression was markedly increased in the roots after NaCl treatment in *Arabidopsis* (Yamada et al., 2011). The expression of *TaSTP12*, *41*, *48*, and *65* was significantly or extremely significantly upregulated in the roots under NaCl-related stress compared to that under the control treatment. However, the STP gene exhibited two opposite expression trends in the roots when PEG6000 was involved in the stress. The *TaSTP41* and *TaSTP65* genes were extremely significantly downregulated, while the *TaSTP12* and *TaSTP48* genes were significant upregulated. Among these four genes, the expression abundance of *TaSTP48* was upregulated up to ten times higher than that in the control, and the other three expression abundances were lower (Fig. 11). Thus, we speculate that *TaSTP48* plays a key role in PEG6000 stress in roots. In *Arabidopsis,* it has been reported that a stress-induced monosaccharide transporter gene, *ESL1,* may cooperate with the sucrose invertase gene in the tonoplast to affect the accumulation of intracellular sugars, thereby regulating the response of plants to abiotic stress (Yamada et al., 2010). According to the latest research, it has been reported that the overexpression of *DsSWEET17* in *Arabidopsis* confers tolerance to salt and oxidative stresses (Zhou et al., 2018). Furthermore, the sugar transporter gene *ERD6* could be upregulated by drought and salt stress in maize (Ma et al., 2009). Drought and high salinity stresses significantly induced the upregulation of *STP13* in *Arabidopsis*. Thus, we speculate that the high expression of *STP13* in the cortex and endodermis may be related to the reabsorption of monosaccharides released from damaged epidermal cells. These findings suggest that the *STP13* gene is involved in an adaptive response to increase cell osmotic pressure or to reduce nutrient loss under abiotic stress (Yamada et al., 2011). Moreover, the proteomics of osmotic stress indicated that water-soluble carbohydrates, including glucose and fructose, were increased in the roots, stems, and leaves in stress plants compared with normal wheat (Ma et al., 2016). Therefore, these results suggest that the *TaSTP* gene may be vital to monosaccharide distribution, regulating the seedling stem and root growth of wheat under drought or salt stress, which is a hypothesis that merits further study.

## Conclusion

We analyzed the sugar transporter genes of wheat at the genome level. Eighty-one TaSTP genes were identified. Gene structure, protein motifs, GO analyses, the expression pattern indicate the conservative and diversified nature of TaSTP genes. Synteny analysis and phylogenetic comparison of TaSTP genes from several different plant species provided valuable insight on the evolutionary characteristics of wheat TaSTP genes. Phylogenetic and gene expression analyses provided important information for the functional analysis of *TaSTP* genes. Our study is a valuable resource for the better understanding of the biological roles of individual TaSTP genes in wheat.

##  Supplemental Information

10.7717/peerj.11371/supp-1 Table S1 Target genes for analysis of expression profiles

10.7717/peerj.11371/supp-2 Table S2 Twenty-two motifs commonly observed in wheat STP proteins

10.7717/peerj.11371/supp-3 Table S3 STP proteins from Arabidopsis thaliana, wheat, rice, brachypodium distachyon, foxtail millet, Manihot esculenta, and Ricinus communis used for phylogenetic analysis

10.7717/peerj.11371/supp-4 Table S4 Bio-linux was used to identify tandem repeat gene pairs and their parameters

10.7717/peerj.11371/supp-5 Table S5 One-to-one orthologous relationships between wheat and other three plant species

10.7717/peerj.11371/supp-6 Table S6 The Ka, Ks and Ka/Ks ratios of TaSTP paralogous pairs

10.7717/peerj.11371/supp-7 Figure S1 Expression profiles of the TaSTP genes

10.7717/peerj.11371/supp-8 Figure S2 Bioinformatic-based protein interaction network analysis of the TaSTPs using STRING 10.5

10.7717/peerj.11371/supp-9 Figure S3 Phenotypic changes of Zhoumai 36 seedlings under different stresses (A-D), (E-H) and (I-L) show the phenotype of plants under NaCl stress, PEG6000 stress and their combined stresses compared with that of control (CK) after 3 days, 6 days and 9 days, respectively.

10.7717/peerj.11371/supp-10 Figure S4 Physiological changes of Zhoumai 36 seedling leaves under NaCl stress, PEG6000 stress and their combined stress treatments A and B show the physiological changes of root length and root width under NaCl stress, PEG6000 stress and their combined stresses compared with that of control after 3 days, 6 days and 9 days, respectively. C and D show the physiological changes of stem length and stem diameter under NaCl stress, PEG6000 stress and their combined stresses compared with that of control after 3 days, 6 days and 9 days, respectively. E and F show the physiological changes of first true leaf length and width under NaCl stress, PEG6000 stress and their combined stresses compared with that of control after 3 days, 6 days and 9 days, respectively. G and H show the physiological changes of second true leaf length and width under NaCl stress, PEG6000 stress and their combined stresses compared with that of control after 3 days, 6 days and 9 days, respectively. Capped lines indicate standard error. * *P* < 0.05; ** *P* < 0.01.

10.7717/peerj.11371/supp-11 Supplemental Information 11 Raw data of Fig S4

10.7717/peerj.11371/supp-12 Supplemental Information 12 Raw data of Figure 11

## Additional Information and Declarations

### Data Availability

The following information was supplied regarding data availability: The raw data are available in the Supplementary Files.
