# Supplementary material for: The Sugar Transporter family in wheat (Triticum aestivum. L): genome-wide identification, classification, and expression profiling during stress in seedlings
Source: PeerJ. 2021 May 4;9:e11371. doi: 10.7717/peerj.11371 (PMC8103919; doi:10.7717/peerj.11371)
Supplement: Table S1 [file peerj-09-11371-s001.docx]

Ta ble S1. Target genes for analysis of expression profiles.

| Primer name | Primer sequences (5´–3´) | Tm (℃) | Product lenth (bp) |
| --- | --- | --- | --- |
| Actin-F | 5´-GGATACACGCTTCCTCATGC-3´ | 58.78 | 128 |
| Actin-R | 5´-CTGACAATTTCCCGCTCAGC-3´ | 59.55 |  |
| TaSTP12-F | 5´-CACCATGTTCGTGCTCTTCG-3´ | 59.56 | 103 |
| TaSTP12-R | 5´-GAAGGGGTTCATCGAGGTCA-3´ | 59.10 |  |
| TaSTP41-F | 5´-CTACTCCTACTATGTAAATG-3´ | 59.06 | 124 |
| TaSTP41-R | 5´-CACATCTTACACACCAGACG-3´ | 58.87 |  |
| TaSTP48-F | 5´-CAGTCGCGAGCCCAACTATTG-3´ | 58.14 | 110 |
| TaSTP48-R | 5´-GCTTATTTTGGGACCGAGTG-3´ | 59.32 |  |
| TaSTP65-F | 5´-CATCTTCGTCATCCTGCTGC-3´ | 58.60 | 115 |
| TaSTP65-R | 5´-GTACTTGGGGTCCTTGGTGA-3´ | 58.04 |  |

F, forward primer; R, reverse primer; Tm, melting temperature.
