# Supplementary material for: The Sugar Transporter family in wheat (Triticum aestivum. L): genome-wide identification, classification, and expression profiling during stress in seedlings
Source: PeerJ. 2021 May 4;9:e11371. doi: 10.7717/peerj.11371 (PMC8103919; doi:10.7717/peerj.11371)
Supplement: Table S2 [file peerj-09-11371-s002.docx]

Table S2. Twenty-two motifs commonly observed in wheat STP proteins.

| Motif | Length | Protein Sequences | Pfam Domain |
| --- | --- | --- | --- |
| 1 | 94 | FICLYVAGFAWSWGPLGWLVPSEIFPLEIRPAGQSINVSVNMLFTFVIAQAFLTMLCHMKFGLFYFFA  GWVVIMTVFIALFLPETKNVPIEEMV | Sugar_tr |
| 2 | 72 | GAALNGAAQNVAMLIVGRMLLGVGIGFANQSVPVYLSEMAPAHLRGMLNIGFQL  MITIGILAAELINYGTNK | Sugar_tr |
| 3 | 41 | AVLIPFFQQLTGINVIMFYAPVLFLTIGFGGDASLMSAVIT | Sugar_tr |
| 4 | 29 | NQYCKYDNQLLQTFTSSLYLAALVSSFFA |  |
| 5 | 36 | KPWGWRLSLGLAAAPALLMTVGGLLLPETPNSLIER | Sugar_tr |
| 6 | 34 | GGKDYPGRLTLFVFFTCVVAATGGLIFGYDIGIS |  |
| 7 | 45 | GAVLLFSTLISIATVDRLGRRKLLISGGIQMIVCQVIVAAILAVK | Sugar_tr |
| 8 | 21 | GVTSMESFLKKFFPDVYHQMH |  |
| 9 | 15 | VWDRHWYWKRFVGDG |  |
| 10 | 21 | DAEFTDMAEASELANTIKHPF |  |
| 11 | 21 | SPVTRNYGRRASIVCGGISFL |  |
| 12 | 15 | GKEEEARAMLRRIRG |  |
| 13 | 11 | LQRKYRPQLTM |  |
| 14 | 15 | SGVGEMPKGYAAAVV |  |
| 15 | 8 | MAGGAVVN |  |
| 16 | 11 | PLEDGWGPGDG |  |
| 17 | 15 | EEEVDQAQTGAGAIA |  |
| 18 | 11 | DHHHNIANGKN |  |
| 19 | 15 | PTPTPDKHADGSLEM |  |
| 20 | 15 | RGDSDLAGADCNVHK |  |
