# Supplementary figures and images for: The Sugar Transporter family in wheat (Triticum aestivum. L): genome-wide identification, classification, and expression profiling during stress in seedlings
Source: PeerJ. 2021 May 4;9:e11371. doi: 10.7717/peerj.11371 (PMC8103919; doi:10.7717/peerj.11371)

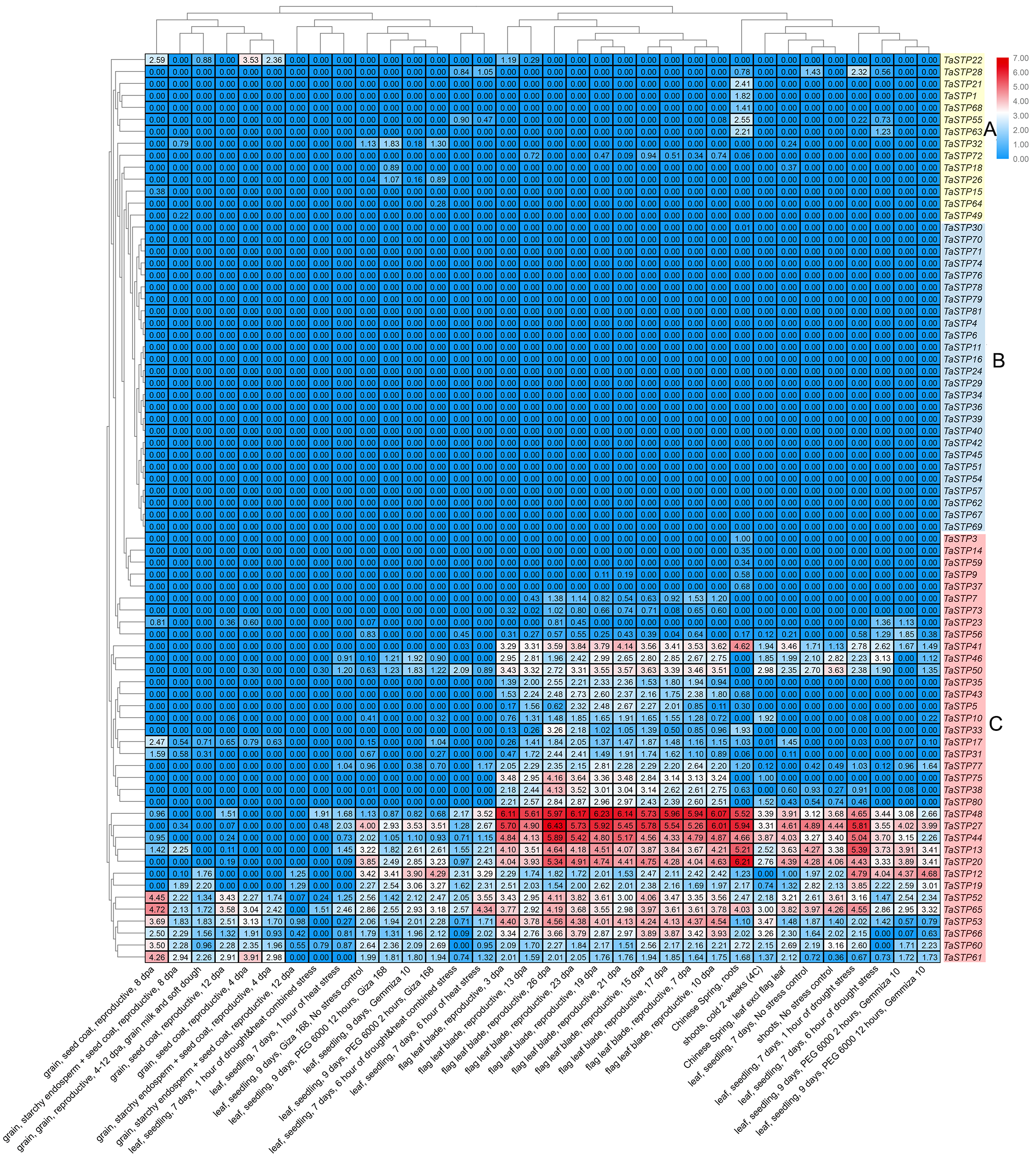

Supplement: Figure S1 [file peerj-09-11371-s007.png]

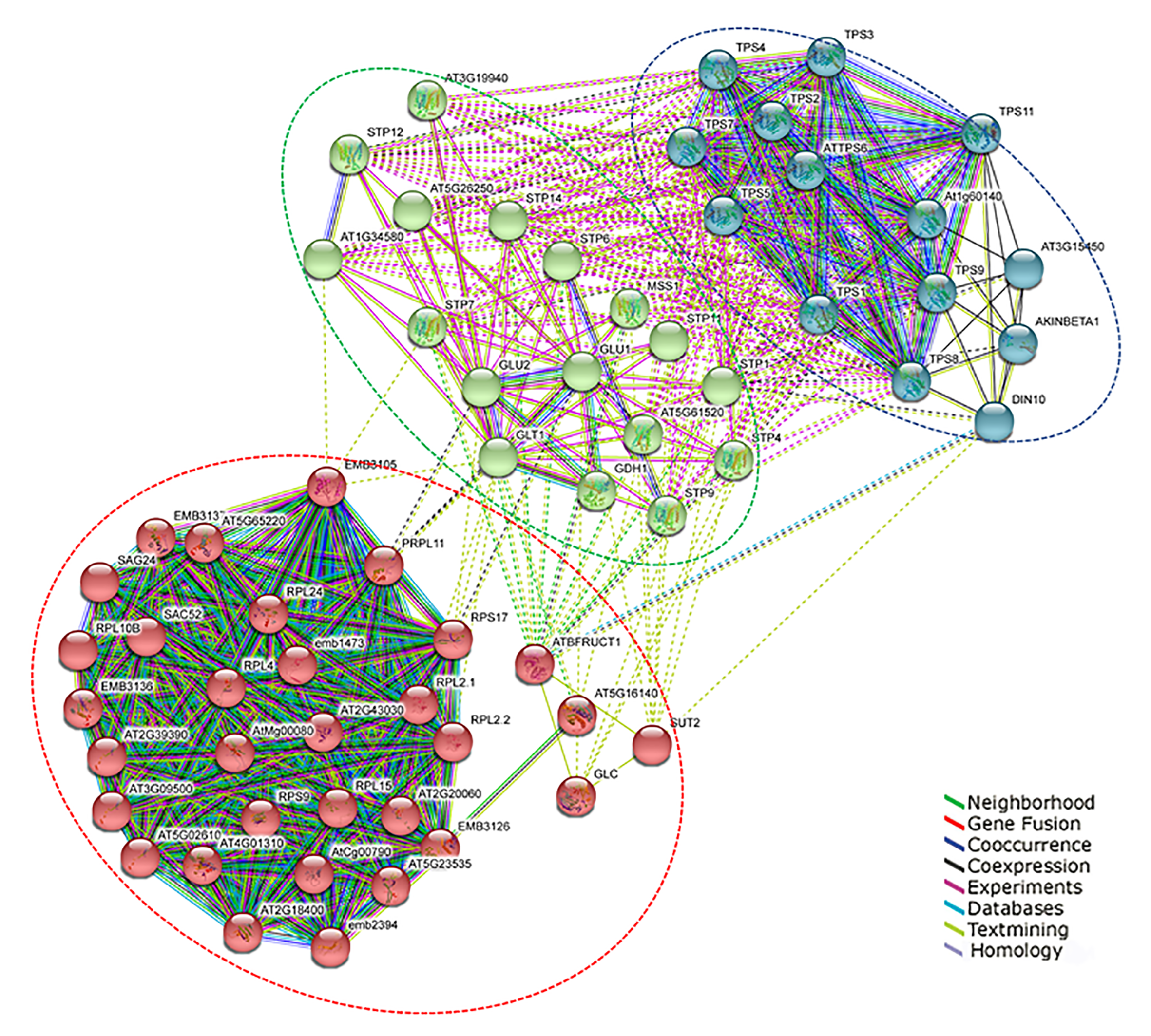

Supplement: Figure S2 [file peerj-09-11371-s008.png]

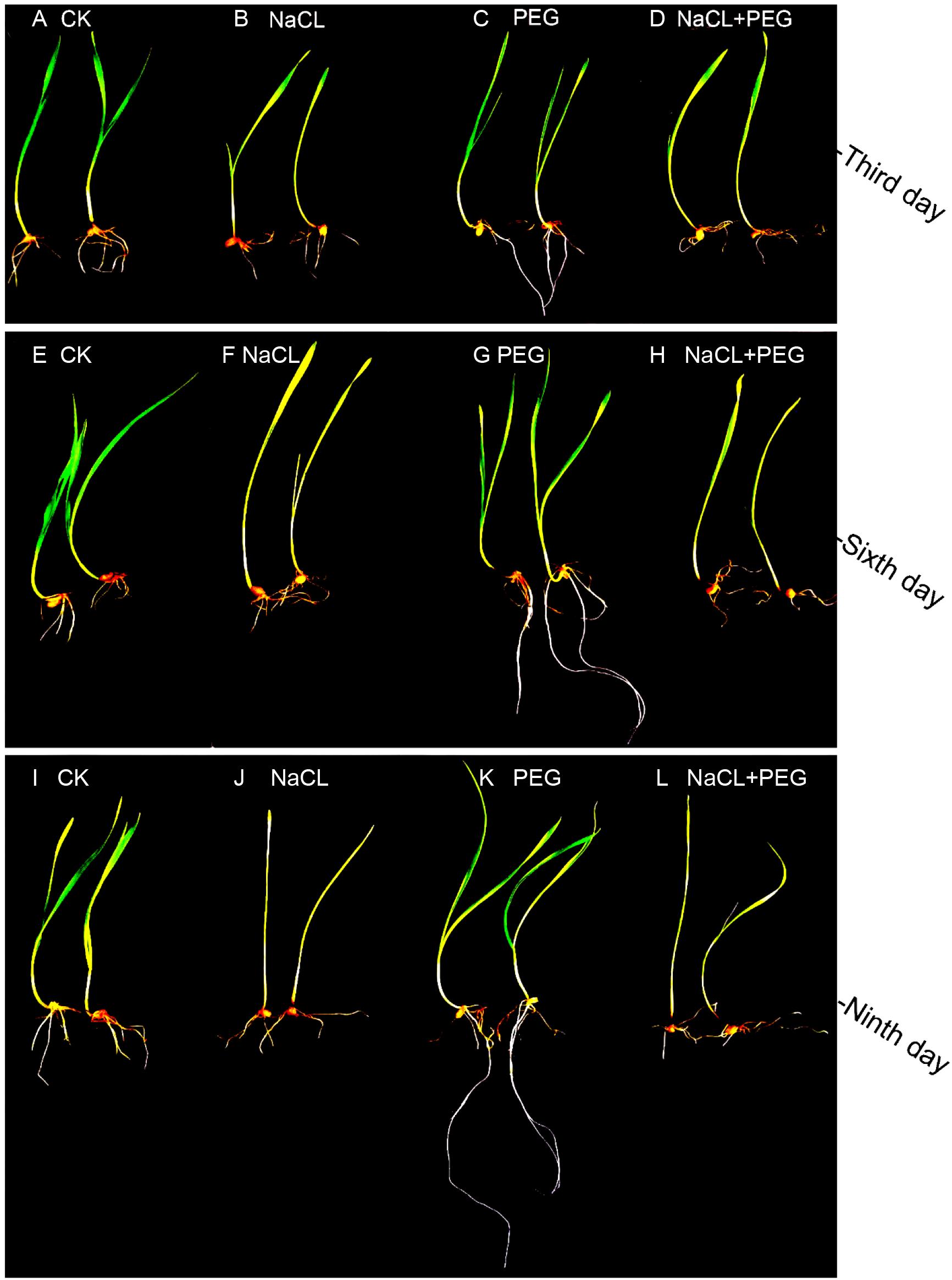

Supplement: Figure S3 — (A-D), (E-H) and (I-L) show the phenotype of plants under NaCl stress, PEG6000 stress and their combined stresses compared with that of control (CK) after 3 days, 6 days and 9 days, respectively. [file peerj-09-11371-s009.png]

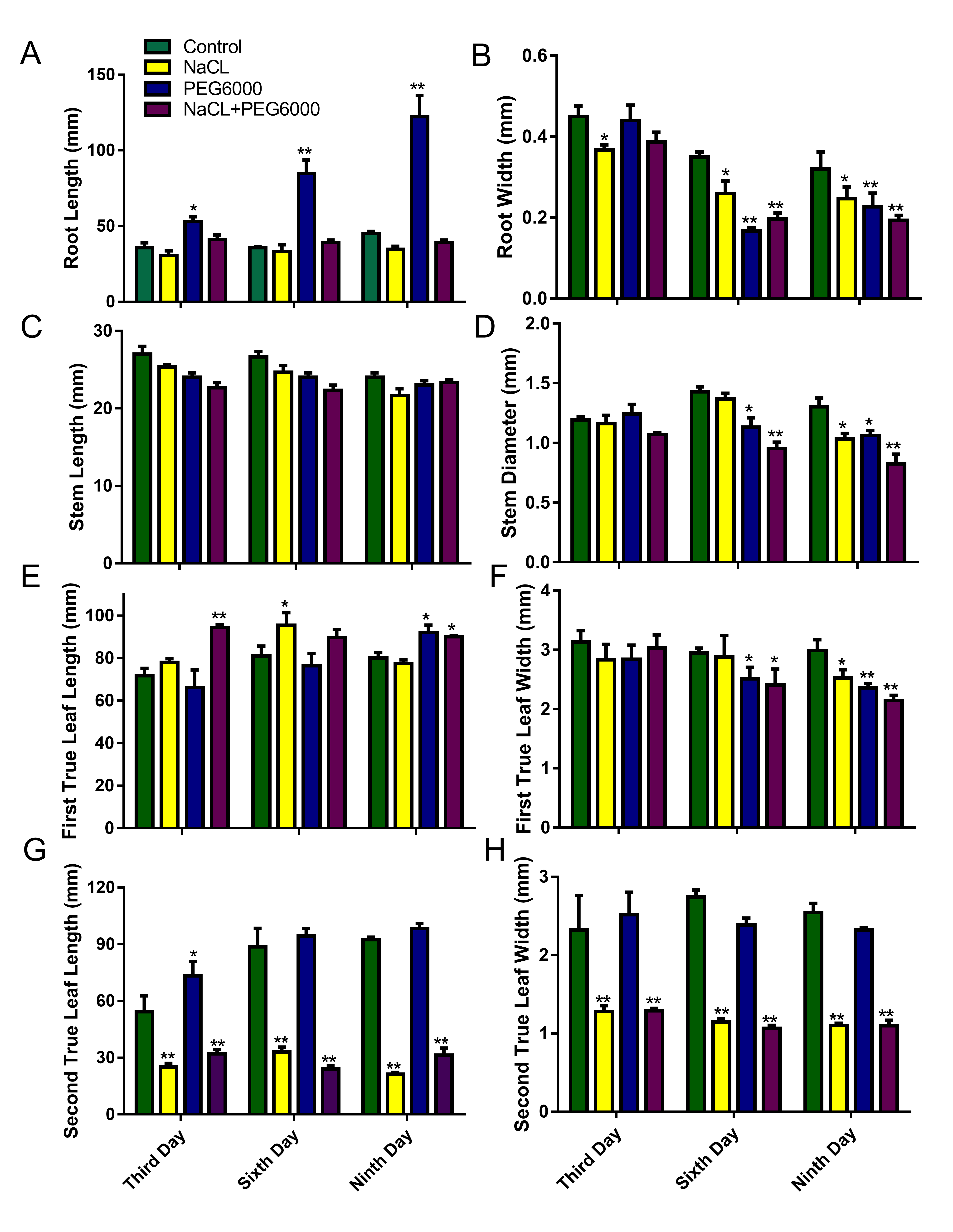

Supplement: Figure S4 — A and B show the physiological changes of root length and root width under NaCl stress, PEG6000 stress and their combined stresses compared with that of control after 3 days, 6 days and 9 days, respectively. C and D show the physiological changes of stem length and stem diameter under NaCl stress, PEG6000 stress and their combined stresses compared with that of control after 3 days, 6 days and 9 days, respectively. E and F show the physiological changes of first true leaf length and width under NaCl stress, PEG6000 stress and their combined stresses compared with that of control after 3 days, 6 days and 9 days, respectively. G and H show the physiological changes of second true leaf length and width under NaCl stress, PEG6000 stress and their combined stresses compared with that of control after 3 days, 6 days and 9 days, respectively. Capped lines indicate standard error. * P < 0.05; ** P < 0.01. [file peerj-09-11371-s010.png]
